# Magnesium enhances the expression of arthroconidia-related genes in *Trichosporon asahii*

**DOI:** 10.1128/spectrum.03459-25

**Published:** 2026-06-15

**Authors:** Keita Aoki, Minenosuke Matsutani, Moriya Ohkuma, Takashi Sugita, Yuuki Kobayashi, Naoto Tanaka, Masako Takashima

**Affiliations:** 1 Laboratory of Yeast Systematics, Tokyo NODAI Research Institute, Tokyo University of Agriculture 13126, Setagaya, Tokyo, Japan; 2 NODAI Genome Research Center, Tokyo University of Agriculture 13126, Setagaya, Tokyo, Japan; 3 Japan Collection of Microorganisms, RIKEN BioResource Research Center 375695 , Tsukuba, Ibaraki, Japan; 4 Department of Microbiology, Meiji Pharmaceutical University 34779, Kiyose, Tokyo, Japan; 5 Department of Molecular Microbiology, Faculty of Life Sciences, Tokyo University of Agriculture 13126, Setagaya, Tokyo, Japan; University of Wisconsin-Madison, Madison, Wisconsin, USA

**Keywords:** arthroconidia, pleomorphism, *Trichosporon asahii*

## Abstract

*Trichosporon asahii* is a pleomorphic basidiomycetous yeast that causes deep-seated mycoses in immunocompromised individuals. *T. asahii* exhibits three morphological types: hyphae, yeasts, and arthroconidia. Hyphal formation is related to virulence and is accelerated by the addition of magnesium. However, the effects of magnesium on gene expression have not been elucidated. In this study, we aimed to investigate whether gene expression is affected by magnesium. RNA sequencing and genetic analyses revealed that magnesium addition increased the expression of genes related to arthroconidia formation in *T. asahii*. We identified 11 magnesium-enhanced expression (*mee*) genes, whose expressions increased more than 5-fold. Deletion of *mee4*, *mee5*, *mee6*, and *mee11* decreased arthroconidia formation. Furthermore, increased *mee6* expression depended on *mee4* and *mee5*, which lie downstream of the TOR pathway, and increased *mee11* expression depended on *mee4*. In addition, *mee11* inhibited the expression of other *mee* genes, and *mee6* inhibited the expression of *mee4*. Overall, we identified a gene expression network related to arthroconidia formation in *T. asahii*, providing insights into the transition from hypha to arthroconidia and enhancing our understanding of fungal pathogenesis.

**IMPORTANCE:**

Magnesium induces cell elongation and changes in organelle distribution in *T. asahii*. In this study, RNA sequencing revealed that magnesium influenced gene expression. Among the genes whose expressions increased more than five-fold following magnesium addition, we identified arthroconidia-related genes whose deletions inhibited arthroconidia formation. Thus, an increase in magnesium cations promotes both hyphal formation and arthroconidia formation in *T. asahii*. This is the first study to elucidate genes related to arthroconidia formation.

## INTRODUCTION

*Trichosporon asahii* is a widely distributed basidiomycetous yeast (1, 2) that shows pleomorphism and causes trichosporonosis, deep-seated mycoses in immunocompromised individuals (3–6). Pleomorphism in *T. asahii* consists of three distinct morphological phenotypes: yeast, hyphae, and arthroconidia (3). Yeast cells are globose or ovoid, whereas hyphae separate into abundant regular, short arthroconidia without lateral conidia or appressoria (7). Arthroconidia are a characteristic form of Trichosporonales (7). Among these morphologies, the hyphal form is associated with epithelial infection (6), whereas the arthroconidial form promotes biofilm formation through enhanced cell adhesion (8). However, the transition between these morphologies is not fully understood.

A recent study reported that hyphal formation is accelerated by magnesium addition in *T. asahii* (9). Magnesium also affects organelle distribution and induces the formation of multiple septa, enlargement of vacuoles, and production of functional lipid droplets in *T. asahii* (9). Similar changes in multi-septa frequency and vacuole volume due to magnesium have been observed in other *Trichosporon* species (10). A comparable effect of magnesium on cell elongation has been described in *Candida albicans*, where it promotes germ tube formation (11). However, whether the supplementation of magnesium affects gene expression in *T. asahii* remains unknown.

In *Saccharomyces cerevisiae*, genomic expression patterns were significantly altered in response to various environmental stresses, including amino acid starvation and nitrogen source depletion (12). Furthermore, in *C. albicans*, hypha-related genes are expressed in response to environmental stimuli (13). Various conditions, including starvation, nutrient availability, osmotic stress, temperature, pH, cell wall damage, pheromones, carbon dioxide, and low oxygen density, have been reported to induce hyphal formation (13). Two major protein kinases—mitogen-activated protein kinase (MAPK) and cAMP-dependent protein kinase—function downstream of these stimuli and regulate the expression of hypha-related genes in *C. albicans* (13). In addition, the target of rapamycin (TOR) signaling pathway negatively regulates hyphal growth in *C. albicans* (14). A similar mechanism of hyphal formation has also been suggested in *T. asahii*: deletion of the MAPK increases hyphal morphology but decreases yeast and arthroconidial morphology (15), while calcineurin-deficient mutations attenuate hyphal formation in both *T. asahii* (16) and *C. tropicalis* (17). However, genes that specifically respond to magnesium have not been investigated.

In this study, we aimed to investigate whether gene expression is altered by magnesium. We identified genes whose expression was highly upregulated by magnesium using RNA sequencing (RNA-seq) and showed that the expression of genes related to arthroconidia formation is enhanced in *T. asahii*.

## RESULTS

### Altered gene expression patterns by magnesium addition

**Fig 1:**
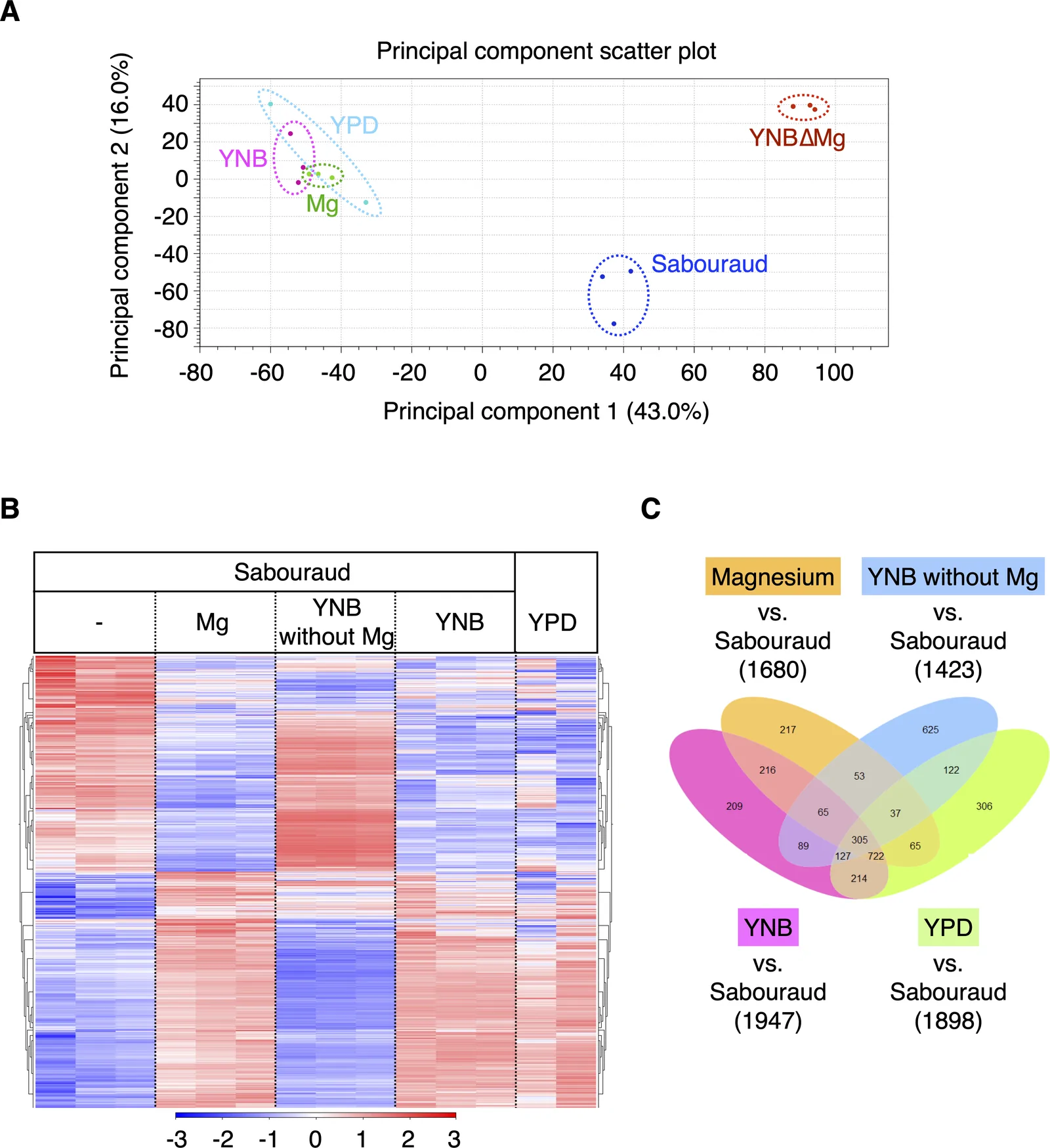
Magnesium alters gene expression patterns in *T. asahii*. (**A**) Principal component scatter plot generated from RNA-seq expression data. (**B**) Heat map analysis showing gene expression levels. Red and blue columns indicate increased and decreased expression, respectively. (**C**) Venn diagram analysis showing the number of genes (in parentheses) whose expression increased by more than two-fold following magnesium addition. Mg, magnesium.

**Fig 2:**
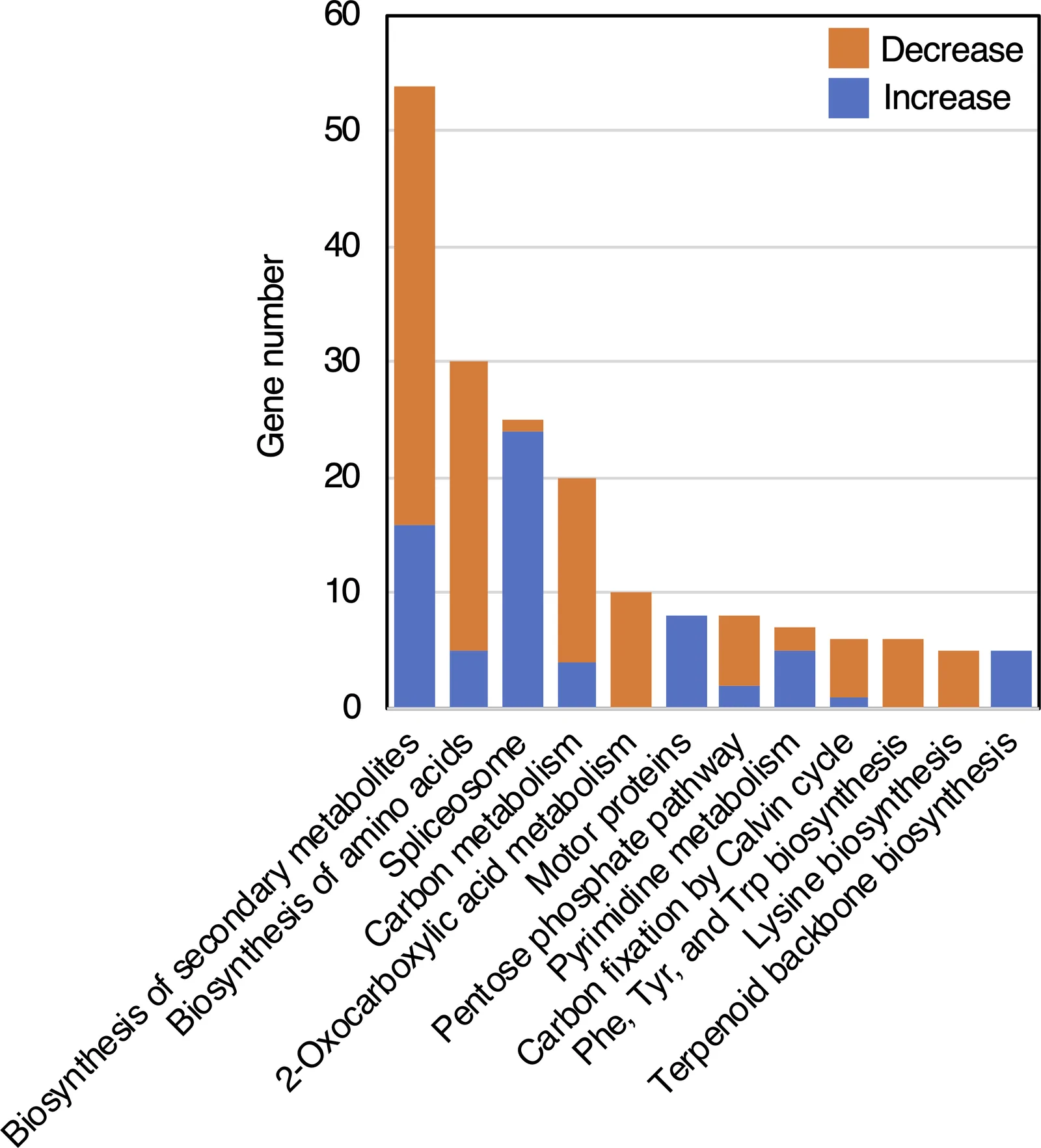
Impacted genes categorized by gene ontology analysis. Of the 722 genes, 94 were classified into 12 pathways using the Kyoto Encyclopedia of Genes and Genomes (KEGG). Some genes are included in multiple pathways. Blue and orange bars indicate the number of genes whose expression increased or decreased, respectively, after magnesium addition.

To examine whether gene expression patterns were influenced by the supplementation of magnesium, we performed RNA-seq using *T. asahii* cells cultured in five different media: YPD broth, Sabouraud broth, and Sabouraud broth supplemented with MgSO_4_ (Sabouraud + Mg), yeast nitrogen base (YNB), and YNB without MgSO_4_ (YNB∆Mg). Principal component analysis (PCA) indicated that gene expression patterns of cells cultivated in YPD broth, Sabouraud + Mg broth, and Sabouraud + YNB broth were similar, and considerably different from those in Sabouraud broth and Sabouraud + YNB∆Mg broth (Fig. 1A). The difference between Sabouraud broth and Sabouraud + YNB∆Mg broth was attributed to YNB components other than MgSO_4_ (Fig. 1A). These differences were also visualized using a heat map, which showed a pattern similar to PCA (Fig. 1B). Genes with increased or decreased expression overlapped among YPD, Sabouraud + Mg, and Sabouraud + YNB media (Fig. 1B). Genes whose expression was altered more than 2-fold were 1,898 in cells cultivated in YPD, 1,680 in Sabouraud + Mg, 1,947 in Sabouraud + YNB, and 1,423 in Sabouraud + YNB∆Mg when compared to Sabouraud alone (Fig. 1C). A Venn diagram showed that among the 8,834 genes predicted in NCBI (https://www.ncbi.nlm.nih.gov/), the expression of 722 genes was commonly altered more than 2-fold in cells cultivated in YPD, Sabouraud + Mg, and Sabouraud + YNB, compared with those in Sabouraud + YNB∆Mg (Fig. 1C). Thus, 722 genes were identified based on their differential expression between cells cultivated in Sabouraud + YNB∆Mg and those in the other three media. Of these, 94 genes were categorized into 12 biological events using the Kyoto Encyclopedia of Genes and Genomes (KEGG) (Fig. 2; Table S1). Most genes were upregulated by magnesium in four biological events: spliceosome, motor proteins, pyrimidine metabolism, and terpenoid backbone biosynthesis (Fig. 2). Notably, 24 of 25 genes in the spliceosome and all eight genes in the motor proteins (including kinesin family, myosin complex, and tropomyosin-like protein) were highly expressed (Table S2).

### Deletion of genes highly expressed due to magnesium addition

**Fig 3:**
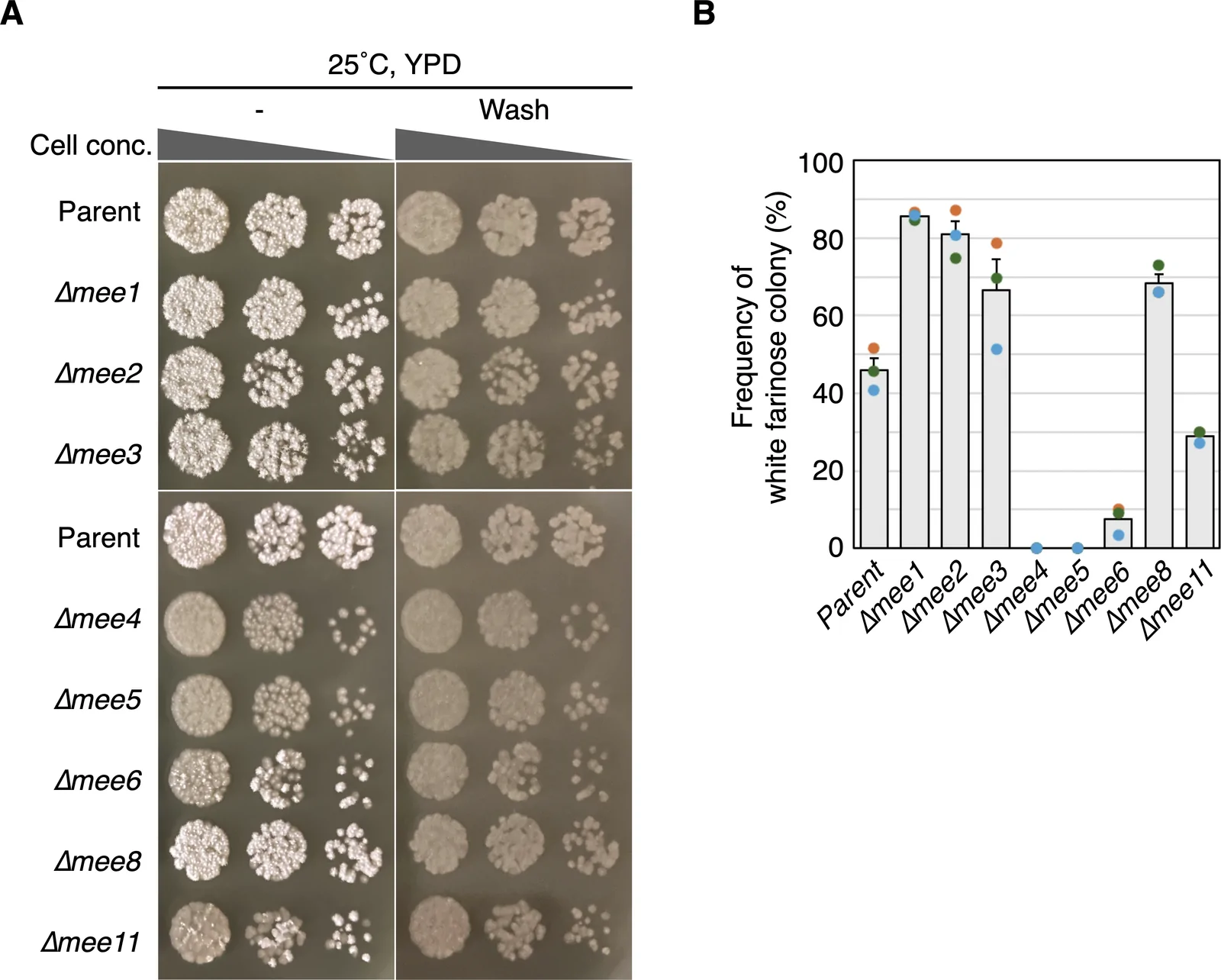
Colony formation phenotypes of deletion mutants. (**A**) Colony formation of deletion mutants observed on YPD agar medium at 25°C. A sequentially diluted cell mixture of 5 µL was spotted onto YPD agar medium (left panels). Colonies on YPD agar medium were washed with water (right panels). (**B**) The cell mixture of 100 µL was spread on YPD agar medium, and cells were incubated for 2 days before the frequency of white farinose colonies was calculated in each mutant.

Among the 722 genes, 15 were upregulated more than 5-fold upon magnesium addition in Sabouraud broth, and 11 of these genes were validated using real-time PCR (Table 1). The 11 genes were designated as magnesium-enhanced expression (*mee*) genes (Table 1). To examine gene function, we deleted these genes and successfully obtained mutants for eight genes: *mee1*, *mee2*, *mee3*, *mee4*, *mee5*, *mee6*, *mee8*, and *mee11* (Table 1). The parental strain produced white farinose colonies on YPD plates that exhibited the powdery surface commonly seen in *Trichosporon* yeasts (7) (Fig. 3A). In contrast, white farinose colonies decreased in *∆mee4*, *∆mee5*, *∆mee6*, and *∆mee11* (Fig. 3A). Hyphal invasion into YPD plates was observed in all strains, as invading cells persisted after washing (Fig. 3A). The frequency of white farinose colonies was examined on YPD plates (Fig. 3B). Notably, white farinose colonies never appeared in *∆mee4* and *∆mee5*, and appeared only partially in *∆mee6* and *∆mee11* (Fig. 3B).

**TABLE 1:** Magnesium-enhanced expression genes

		Fold changes (*P*-values)		
Locus_tag	Genes	RNA-seq	RT-PCR	Putative function
A1Q1_04050	*mee1*	14.4 (1.5 × 10^−2^)	8.3 (4.9 × 10^−6^)	Hypothetical
A1Q1_07651	*mee2*	9.0 (1.5 × 10^−13^)	2.9 (9.2 × 10^−3^)	D-galactonate transporter
A1Q1_03805	*mee3*	7.7 (8.3 × 10^−4^)	4.3 (2 × 10^−3^)	Hypothetical
A1Q1_01385	*mee4*	6.1 (9.7 × 10^−12^)	3.4 (2.9 × 10^−5^)	Hypothetical
A1Q1_02761	*mee5*	6.1 (2.2 × 10^−3^)	8.9 (3.1 × 10^−4^)	Nop14-like family
A1Q1_03141	*mee6*	6.1 (5.2 × 10^−24^)	2.2 (3.6 × 10^−5^)	Arrestin-C
A1Q1_05074	*mee7*	5.5 (2.3 × 10^−19^)	3.1 (4 × 10^−5^)	DASH complex subunit Dad4
A1Q1_07116	*mee8*	5.3 (2.7 × 10^−28^)	4.1 (5.8 × 10^−6^)	NAD(P)-dependent dehydrogenase
A1Q1_05377	*mee9*	5.1 (1.2 × 10^−13^)	6.3 (8.7 × 10^−6^)	Mandelate racemase
A1Q1_00560	*mee10*	5.1 (6.2 × 10^−26^)	3.2 (5.4 × 10^−6^)	Hypothetical
A1Q1_01176	*mee11*	5.1 (5.6 × 10^−13^)	9.9 (4.6 × 10^−4^)	Hypothetical

### Arthroconidial phenotypes in the parental strain and deletion mutants

**Fig 4:**
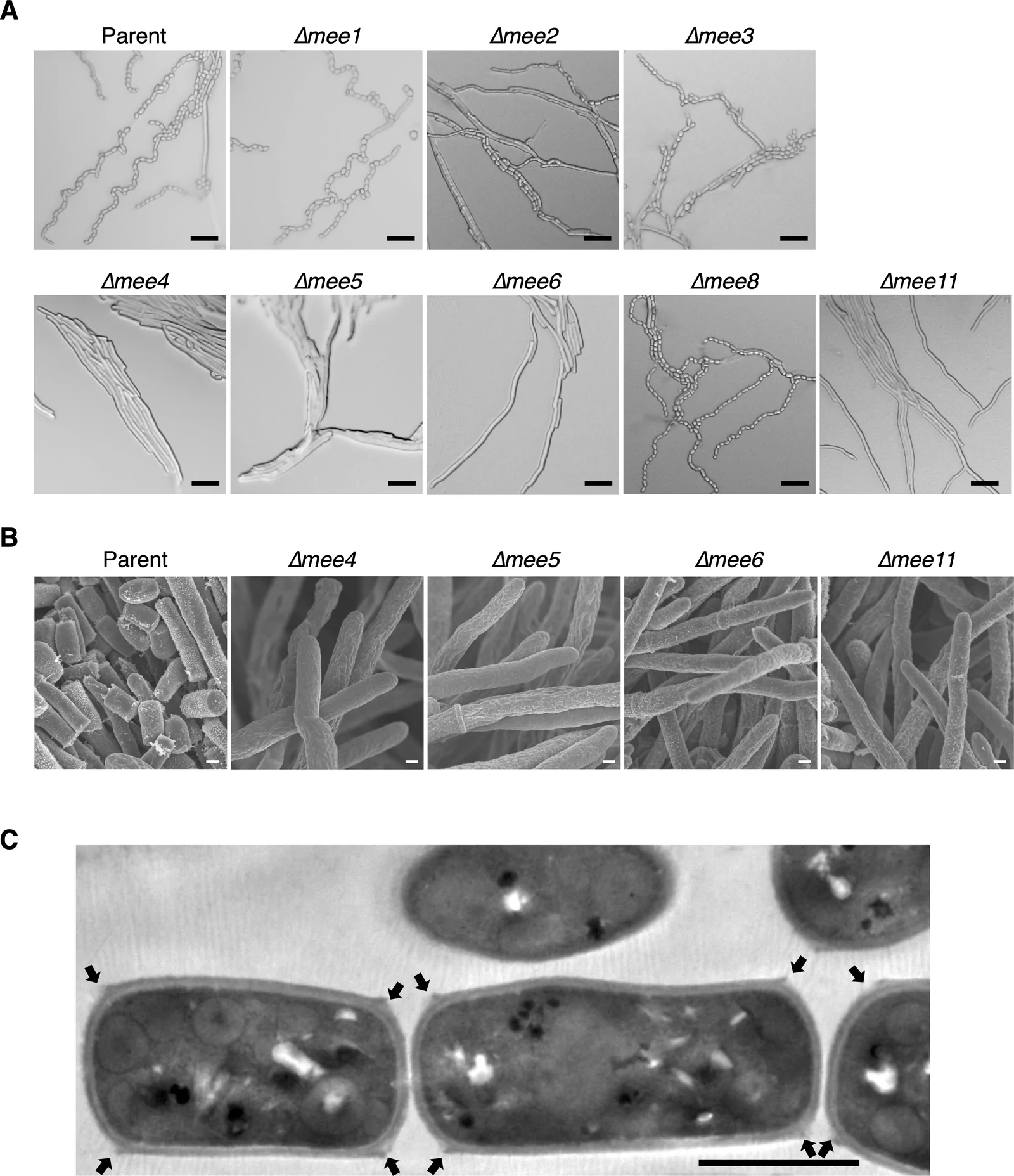
Arthroconidia formation is impacted by gene deletions. (**A**) Phenotypes of mutant cells grown on YPD agar medium observed under a fluorescence microscope. Scale bar = 10 µm. (**B**) Phenotypes of mutant cells observed by SEM. Scale bar = 1 µm. (**C**) Cylindrical and spherical parental strain cells observed by TEM. Arrows indicate gaps in cell wall structure. Scale bar = 2 µm.

We visualized colonies of the parental strain and the deletion mutants on YPD plates. Parental strain cells frequently formed arthroconidia, linked like beads around colony edges (Fig. 4A). Similar arthroconidia were observed in *∆mee1*, *∆mee2*, *∆mee3*, and *∆mee8* (Fig. 4A). In contrast, arthroconidia were rarely observed, and hyphae were abundant in *∆mee4*, *∆mee5*, *∆mee6*, and *∆mee11* (Fig. 4A). Scanning electron microscopy (SEM) revealed cylindrical, regular, and short cells, characteristic of arthroconidia, in the parental strain, whereas hyphae predominated in *∆mee4*, *∆mee5*, *∆mee6*, and *∆mee11* (Fig. 4B). Transmission electron microscopy (TEM) further showed cylindrical cells with gaps in the cell wall at the edges that showed discontinuities in the cell wall (Fig. 4C). No gaps were observed in spherical cells of *T. asahii*. These results indicate that arthroconidia increase in white farinose colonies in the parental strain and that *mee4*, *mee5*, *mee6*, and *mee11* are required for cylindrical arthroconidia formation in *T. asahii*.

### Putative functions and commonality of arthroconidia-related genes

**Fig 5:**
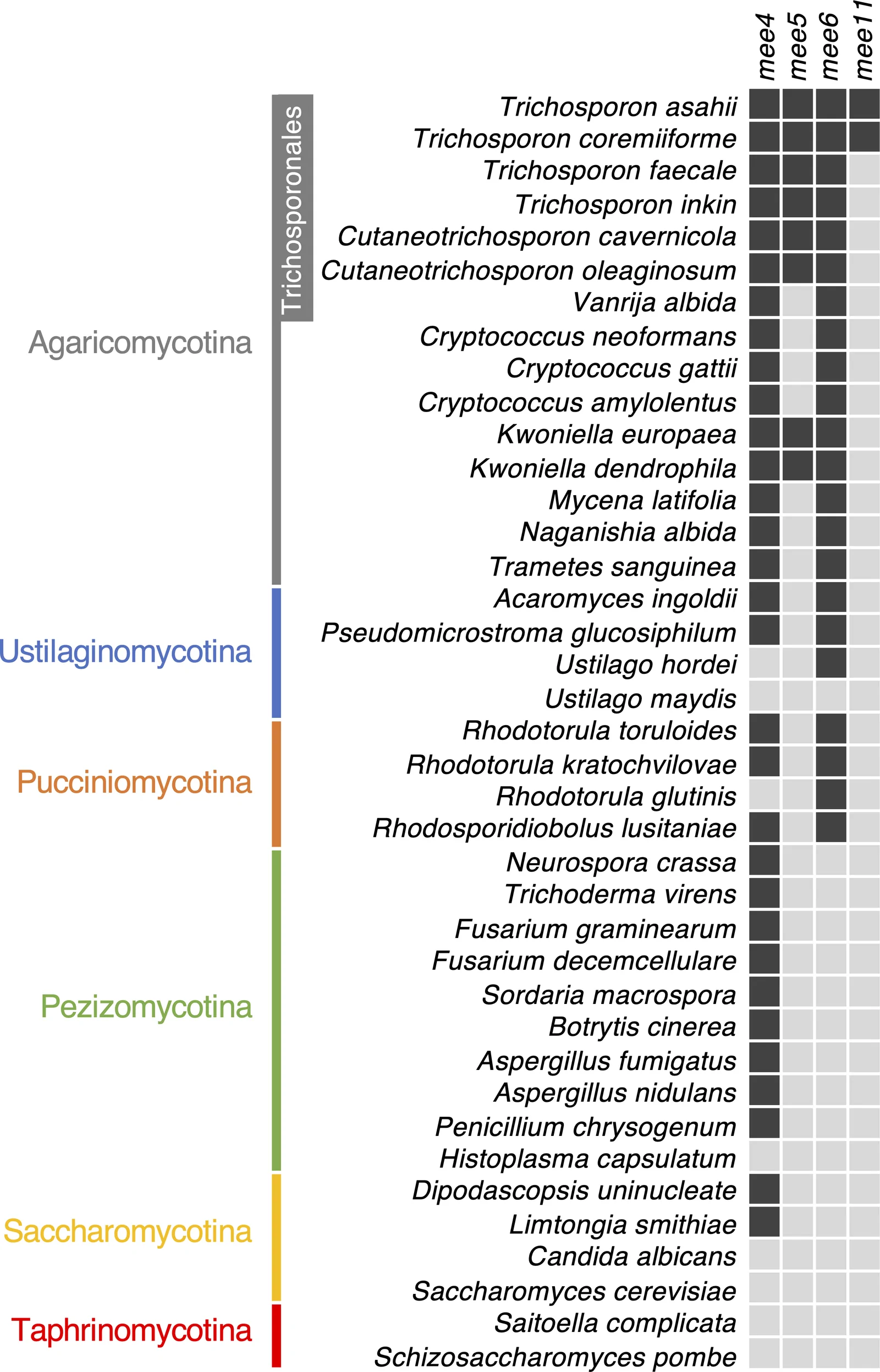
Distribution of *mee* genes among Dikarya. Presence (deep gray) or absence (light gray) of *mee* genes is represented among selected species across Agaricomycotina, Ustilaginomycotina, Pucciniomycotina, Pezizomycotina, Saccharomycotina, and Taphrinomycotina.

The putative functions of these genes were examined using the NCBI database (https://www.ncbi.nlm.nih.gov/). Mee5 has a Nop14-like family motif (pfam04147) that is specific for the nucleolar protein Nop14 (18), a component of the small subunit (SSU) processome complex involved in 40S ribosomal subunit maturation and nuclear export (19) (Table 1). Mee5 also contains an Abf1 promoter-like sequence (20) (ATCACTCCCCACGG) located 217 bp upstream, which regulates the expression of ribosomal proteins. Four genes with the Nop14-like family motif are present in the *T. asahii* genome, and *mee5* expression was only affected by magnesium. Mee5 homologs are present only in Agaricomycotina and are notably abundant in Trichosporonales other than *Vanrija albida* (Fig. 5). Mee6 has an Arrestin C-terminal domain (smart01017), regulated by G protein-coupled receptors (GPCRs) required for heterotrimeric G protein activation (21) (Table 1). Four genes with the Arrestin C-terminal domain are present in the *T. asahii* genome, and *mee6* expression was only affected by magnesium. Mee6 homologs are present in Agaricomycotina, Ustilaginomycotina, and Pucciniomycotina (Fig. 5). Mee4 and Mee11 are hypothetical proteins: Mee4 homologs are present in Agaricomycotina, Ustilaginomycotina, Pucciniomycotina, Pezizomycotina, and Saccharomycotina (Fig. 5), whereas Mee11 homologs are unique to *T. asahii* and *T. coremiiforme* in Agaricomycotina (Fig. 5).

### Increased expression of *mee* genes due to magnesium addition is interdependent

**Fig 6:**
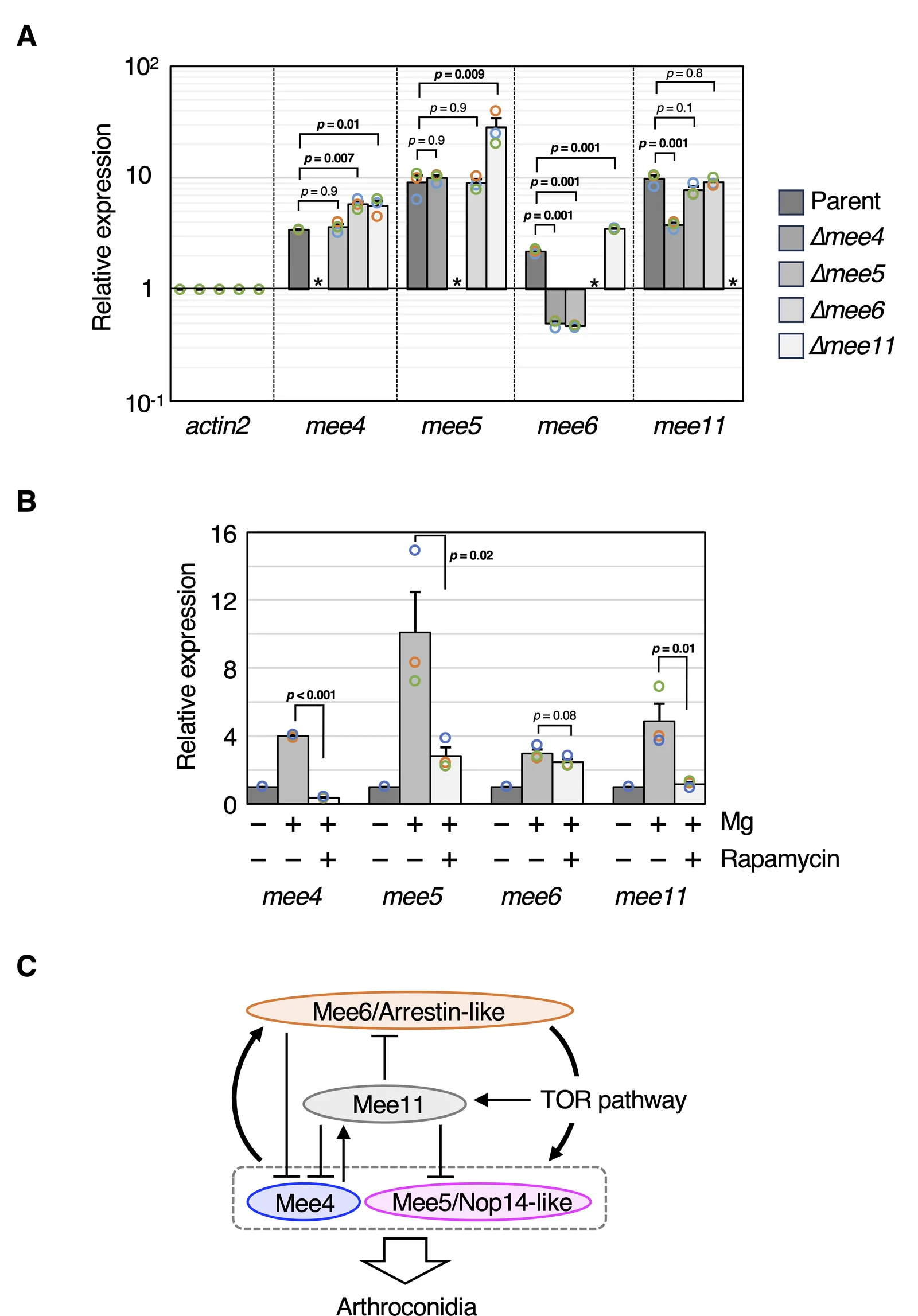
Putative gene expression network upstream of arthroconidia formation. (**A**) Real-time PCR experiments examining expression of target genes in parental, *∆mee4*, *∆mee5*, *∆mee6*, and *∆mee11* strains. Each value on the vertical axis represents the fold increase in expression in Mg-supplemented samples relative to YNB∆Mg-supplemented samples, expressed on a logarithmic scale. All values are normalized to *actin2* expression. Asterisks indicate that experiments were not performed due to gene deletion. (**B**) Real-time PCR experiments examining expression of *mee4*, *mee5*, *mee6*, and *mee11* with or without rapamycin treatment. Each value on the vertical axis represents the expression levels in Mg-supplemented samples with or without rapamycin relative to YNB∆Mg-supplemented samples. All values are presented in Table S6. (**C**) Putative expression network among *mee* genes upstream of arthroconidia formation. Mee4 and Mee5 are surrounded as a group by a dotted line. Expression relationships between Mee6 and the group are represented by thick curved arrow lines. *P*-values significant at *P* < 0.05 were in bold. Mg, magnesium. YNB, yeast nitrogen base.

To test whether magnesium-induced *mee* gene expression was interdependent, real-time PCR was performed using total RNA purified from the parental strain, *∆mee4*, *∆mee5*, *∆mee6*, and *∆mee11. Mee6* expression was significantly decreased in *∆mee4* (*P* = 0.001) and *∆mee5* (*P* = 0.001) compared with the parental strain (Fig. 6A). *Mee11* expression was also decreased in *∆mee4* (*P* = 0.001). In contrast, *mee4* (*P* = 0.01), *mee5* (*P* = 0.009), and *mee6* (*P* = 0.001) expression increased in *∆mee11* compared with the parental strain (Fig. 6A). *Mee4* expression was also increased in *∆mee6* (*P* = 0.007). These findings suggest that *mee6* expression is downstream of *mee4* and *mee5* and *mee11* expression is downstream of *mee4* (Fig. 6C). *Mee11* negatively regulates *mee4*, *mee5*, and *mee6*, whereas *mee6* negatively regulates *mee4* (Fig. 6C).

### Rapamycin affects the expression of arthroconidia-related genes

To determine whether *mee* gene expression is linked to the TOR pathway, real-time PCR was performed on rapamycin-treated cells. Expression of *mee4*, *mee5*, and *mee11*—which were upregulated by magnesium—was decreased by rapamycin to 0.09-fold (*P* < 0.001), 0.3-fold (*P* = 0.02), and 0.2-fold (*P* = 0.01), respectively, (Fig. 6B). In contrast, *mee6* expression, which was also increased by magnesium addition, was unaffected by rapamycin (*P* = 0.08) (Fig. 6B). These findings suggest that *mee4*, *mee5*, and *mee11*, and not *mee6*, are downstream of TOR signaling (Fig. 6C). Collectively, these data identify a gene expression network that regulates arthroconidia formation (Fig. 6C).

## DISCUSSION

In this study, we identified magnesium-enhanced expression (*mee*) genes whose expression increased more than 5-fold after magnesium addition in *T. asahii*. Among them, we identified four genes—*mee4*, *mee5*, *mee6*, and *mee11*—as arthroconidia-related genes, as deletion of any of these genes weakened arthroconidia formation in *T. asahii*. Real-time PCR experiments showed that *mee6* expression was downstream of *mee4* and *mee5,* and *mee11* expression was downstream of *mee4*, while *mee11* attenuated the expression of *mee4*, *mee5*, and *mee6*, and *mee6* weakened the expression of *mee4*. Furthermore, *mee4*, *mee5*, and *mee11* lay downstream of the TOR pathway. Together, we identified a novel gene expression network upstream of arthroconidia formation in *T. asahii*.

Magnesium accelerates hyphal formation in *T. asahii* (9). Our study showed that magnesium affected approximately 10% of the total gene expression. Biological events involving spliceosome, motor proteins, pyrimidine metabolism, and terpenoid backbone biosynthesis seemed to be upregulated by magnesium; however, the mechanism remains hypothetical. Notably, genes involved in the spliceosome were broadly influenced, and their expression was increased by magnesium, suggesting that alterations in mRNA and protein sequences may affect hyphal characteristics in *T. asahii*. An increase in spliceosome activity may have pleiotropic effects on cell morphology. In addition, increased expression of kinesin family, myosin complex, and tropomyosin-like proteins may promote cell elongation. Moreover, arthroconidia-related genes were highly expressed in response to magnesium addition. Therefore, magnesium addition may simultaneously affect the expression of genes related to both hyphal and arthroconidial formation in *T. asahii*.

Spliceosome-related genes whose expression increased with magnesium addition in *T. asahii* include A1Q1_00233, which belongs to the BUD31 family. Repression of BUD31 led to decreased filamentation in *C. albicans*, possibly due to altered intron retention (22). Thus, it may be common that alteration in splicing activity affects hyphal growth. Furthermore, a relationship between the spliceosome and motor proteins has been reported in *Ustilago maydis*. In *U. maydis*, Num1, a homolog of SPF27 that is a component of an integral subunit of the spliceosome, is known to be required for polarized hyphal growth (23). Num1 interacts with not only spliceosome components Prp19 and Cef1 but also the motor protein Kinesin I and factors involved in endosome-trafficking processes. Thus, Num1 has dual functions for splicing in the nucleus and Kin I-dependent intracellular trafficking processes in the cytoplasm. Therefore, the BUD31 homolog might contribute to kinesin dynamics in *T. asahii*.

Magnesium-induced pyrimidine metabolism might contribute to hyphal formation through synthesis of uridine diphosphate (UDP) and cytidine diphosphate (CDP), because A1Q1_04436, whose expression increases with magnesium addition, is a candidate homolog of UMP/CMP kinase (EC 2.7.4.14). Nucleotide diphosphate sugars are donors for the production of carbohydrate chains in glycosylation necessary for cell wall glucan synthesis (24). In *Aspergillus nidulans*, loss of UDP-galactofuranose causes abnormal hyphal morphology (25). Therefore, magnesium might affect glycosylation, thereby influencing cell wall synthesis and hyphal growth in *T. asahii*. While on terpenoid backbone biosynthesis, synthesis of farnesol might increase with magnesium and relate to hyphal formation. This is because inhibition of farnesylation of CENP-E, a centromere protein, results in the alteration of the microtubule-centromere interaction and attenuates microtubule extension (26, 27).

Arthroconidia were abundant, regular, and short cells linked together directionally on YPD agar medium, consistent with a previous report (7), and might mainly form white farinose colonies. Cylindrical cells of arthroconidia, observed by SEM, may be produced by fragmentation of hyphae rather than by budding in *T. asahii*. TEM images showed that these cylindrical cells had gaps in the cell wall at the cell edges, probably representing remnants of hyphal septa. Moreover, deletion of arthroconidia-related genes did not affect hyphal formation on YPD agar medium. These observations strongly support the hypothesis that septate hyphae give rise to arthroconidia in *T. asahii*.

Based on the NCBI database (https://www.ncbi.nlm.nih.gov/), Mee5 and Mee6 were inferred to be proteins with a specific motif for Nop14 and Arrestin-C in *T. asahii*, respectively. However, they are not conserved in ascomycetes and are therefore thought to be the genes that have evolved specifically in basidiomycetes. Commonality data suggest that *mee4*, *mee5*, and *mee6* are collaboratively associated with arthroconidia formation in Trichosporonales. The lack of *mee5* homolog in *Varija albida* may be related to the fact that it does not produce arthroconidia although it belongs to Trichosporonales (28). However, *Kwoniella* yeasts may have arthroconidia-form because homologs of the three genes are conserved in the genomes.

Our genetic data indicated that Mee6 expression was downstream of Mee5 and that Mee5 expression was in turn downstream of the TOR pathway. The TOR pathway is known to be associated with arrestin (29). Therefore, there may be a regulatory mechanism involving Mee5 and Mee6 in *T. asahii*. Mee11 inhibited the expression of arthroconidia-related genes. The functions of Mee11 remain unidentified but may represent *T. asahii* and *T. coremiiforme*-specific phenotypes, as *mee11* is unique to these species. While Mee6 inhibited the expression of *mee4*, which may be more common among yeasts with arthroconidia, because homologs of the two genes are broadly conserved in basidiomycetes. Mee4 showed a similar phenotype as that of Mee5 in *T. asahii*. However, Mee4 homologs were present even in ascomycetes, which was different from that of Mee5. Among the arthroconidia-related genes, Mee4 and Mee5 are thought to act the nearest to arthroconidia formation, as white farinose colonies were never observed in *∆mee4* and *∆mee5*. Nop14 is a nucleolar protein and a component of the SSU processome (19). Therefore, Mee5 potentially functions as a component of the SSU processome in *T. asahii* hyphae. In *S. cerevisiae*, defects in the SSU processome inhibit progression through the G1 phase (30). Similarly, Mee5 may be related to G1 phase progression in *T. asahii* hyphae; therefore, deletion of *mee5* may result in impaired cell division required for arthroconidia formation.

In summary, we identified arthroconidia-related genes and a potential gene expression network upstream of arthroconidia formation in *T. asahii*. However, this study did not differentiate between septa formed in the hyphae and those that lead to the formation of arthroconidia. Further studies are required to elucidate the mechanism underlying arthroconidia formation at the protein level.

## MATERIALS AND METHODS

### Strains and media

JCM 2466 was provided by the Japan Collection of Microorganisms, RIKEN BioResource Research Center (Tsukuba, Japan). MPU 129 *∆ku70* was previously reported (8, 24). Sabouraud (4% wt/vol glucose and 1% wt/vol bacto peptone), Sabouraud-hp (4% wt/vol glucose and 1% wt/vol hipolypepton [Shioya-MS Co., Ltd., Hyogo, Japan]), and YPD (2% wt/vol glucose, 1% wt/vol yeast extract, and 2% wt/vol bacto peptone) media were used. Yeast nitrogen base (Becton, Dickinson, and Company, Franklin Lakes, MD, USA) and 4.15 mM MgSO_4_ (KANTO CHEMICAL Co., Inc., Tokyo, Japan) were added to the Sabouraud broth. The concentration of MgSO_4_ was determined in the previous report as it fully induced hyphal growth in *T. asahii* (9). To experimentally test the effect of YNB components other than MgSO_4_, a mixture of YNB components other than MgSO_4_ (YNB∆Mg) was prepared by self and used for the study. To purify total RNA for RNA-seq and qPCR experiments, the broth of each medium was used. To observe phenotypes of colonies and cell morphologies, agar plates of each medium were used; 2% wt/vol agar was added to solid medium.

### Purification of total RNA

Total RNA was purified from JCM 2466 cells cultivated in the log phase in five types of 16 mL media: YPD broth, Sabouraud broth, and Sabouraud broth supplemented with MgSO_4_, YNB, or YNB without MgSO_4_ using a NucleoSpin RNA Plant and Fungi Kit (MACHEREY-NAGEL, Düren, Germany). Three replicates were used for conditions without YPD, and two replicates were used for YPD. To disrupt the cells, the cells with PFL and PFR buffers were added to a MN Beads Tubes Type A (MACHEREY-NAGEL) and vortexed vigorously twice for 15 s on ice.

### RNA-seq

The NEBNext poly(A) mRNA Magnetic Isolation Module (New England Biolabs, Ipswich, MA, USA) was used to purify poly(A) mRNA from *T. asahii*. The NEBNext Ultra II Directional RNA Library Prep Kit for Illumina (New England Biolabs) was used to remove rRNA, purify mRNA, and construct the mRNA library. NEBNext multiplex oligos from Illumina were used to provide adaptors and primers for the library. Sequencing was performed using NextSeq 500 (New England Biolabs) with single-end design. Average length and number of reads for each condition was followed; Sabouraud (340 bp, 17,356,357 reads), Sabouraud + Mg (329 bp, 16,302,470 reads), Sabouraud + YNB (332 bp, 17,956,720 reads), Sabouraud + YNB∆Mg (343 bp, 20,101,619 reads), and YPD (325 bp, 15,754,243 reads). RNA-seq data were analyzed by CLC genomic work bench (QIAGEN, Hilden, Germany). A total of 722 genes affected via magnesium addition were classified by DAVID (the Database for Annotation, Visualization, and Integrated Discovery) (NIH, Bethesda, MD, USA), and KEGG pathways were selected.

### Real-time PCR

Total RNA was treated with DNase I (Thermo Fisher, Waltham, MA, USA) for 30 min at 37°C to remove genomic DNA contamination. cDNA was synthesized from DNase-treated RNA using PrimeScript RT Master Mix (TAKARA, Shiga, Japan). For the reverse transcription reaction, RNA mixture was incubated for 15 min at 37°C. Real-time PCR was performed using TB Green Premix Ex Taq III (TAKARA) on Thermal Cycler Dice Real-Time System III (TAKARA). Primers for real-time PCR were designed using the Primer3Plus website (https://www.primer3plus.com/index.html) such that the amplified products were 100–150 bp in length and are listed in Table S3. Reactions were carried out in duplicate for each sample in 25 µL volume. Real-time PCR experiments were repeated three times for each sample, and average values are shown. All values determined using Real-time PCR were normalized by *actin2* expression. The cycling conditions were as follows: 95°C 30 s for initial denaturation, followed by 40 cycles of 95°C 5 s and 60°C 30 s, and a final dissociation cycle.

### Electroporation

A modified version of the transformation method described by Matsumoto et al. (31, 32) was used. Briefly, MPU129 *∆ku70* cells were cultured on Sabouraud-hp agar medium containing 50 µg/mL G418 (Enzo, Farmingdale, NY, USA) for 24 h at 25°C. The cells were suspended in 2 mL PBS on Sabouraud agar medium, and the suspension was filtered through a 40-µm cell strainer (AS ONE corporation, Osaka, Japan). The cell suspension (0.15 mL) was plated on Sabouraud agar medium and cultured for 18 h at 25°C. The cells were again suspended in 2 mL PBS, filtered through a 40 µm cell strainer, and transferred into a 1.5 mL tube. The cells were washed four times with ice-cold sterile water and suspended in 50 µL of 1M sorbitol. The cell suspension was mixed with a Nourseothricin-resistant gene fragment containing 5′ and 3′ untranslated region of the target gene. A nourseothricin-resistant gene was amplified from FYP4244 plasmid (National Bio-Resource Project (NBRP), Osaka, Japan) using the following primer set: 5′-CGACATGGAGGCCCAGAATACCCT-3′ and 5′-CAGTATAGCGACCAGCATTCACAT-3′. The cell mixture was incubated for 15 min at 4°C, transferred into a cuvette at a 0.2 cm gap, and pulsed at 1.8 kV for 5 ms using a Gene Pulser Xcell (BioRad, Hercules, CA, USA). The cell mixture was immediately mixed with 0.5 mL YPD broth supplemented with 0.6 M sorbitol and incubated for 3 h at 25°C. The culture was centrifuged at 9,060 × *g* for 5 min at 20°C, and the supernatant was removed. The cell pellet was suspended in 100 µL PBS and plated onto a Sabouraud agar medium supplemented with 350 µg/mL Nourseothricin (Jena Bioscience, Thuringia, Germany). To verify the replacement of each target gene with the nourseothricin resistance gene in the *T. asahii* genome, colony PCR was performed using three primer sets: F, R, and K. (Table S4). All PCR experiments were performed using KOD One PCR enzyme (TOYOBO, Osaka, Japan) and the 5′ and 3′ untranslated regions of each target gene were amplified using the primers listed in Table S4.

### Spot assay

Cells were cultivated to log phase in YPD broth and diluted to optical density (OD_660_) = 0.1 in sterile water. The cell mixture was sequentially diluted to 5^−3^, 5^−4^, and 5^−5^ before spotting 5 µL in three places onto YPD agar medium. In addition, the cell mixture was diluted to 5^−5^ of which 100 µL was spread on YPD agar medium, and the cells were incubated for 2 days at 25°C. The number of white farinose colonies was counted three times independently and their average frequency was calculated.

### Slide culture observation

Cells were cultivated on YPD agar medium at 25°C. For observation, YPD agar medium was prepared on a microscope glass slide with removable two-well chambers (Matsunami, Osaka, Japan). A small number of cells were placed on the YPD agar medium and covered with a cover glass (Matsunami). The chamber was incubated for two days at 25°C before observation under a microscope (OLYMPUS BX53, Olympus, Tokyo, Japan), equipped with a U-FDICT mirror unit at 40× magnification. Cells around colony edges were observed.

### Electron microscopy observation

Cells of parent, *∆mee4*, *∆mee5*, *∆mee6*, and *∆mee11* strains were cultivated on YPD agar medium for 4 days at 25°C. Cells were cut into 5 mm agar squares and fixed in 2% glutaraldehyde in 0.1% phosphate buffer (pH 7.4). Observations using scanning and transmission electron microscopy were performed by the Hanaichi UltraStructure Research Institute, Co., Ltd. (Aichi, Japan).

### Search for homologs

Homologs are examined using the full amino acid sequence of each *mee* gene under the condition: database is non-redundant protein sequence, algorithm is blastp, expect threshold in general parameters is 0.05, and matrix in scoring parameters is BLOSUM62. Genes with E-value <0.001 were considered homologs. Homolog numbers of Mee4, Mee5, Mee6, and Mee11 were 1,297, 32, 706, and 1 in the blastp, respectively. In addition, homologs in *T. coremiiforme*, *T. faecale*, and *T. inkin* were examined by OrthoFinder software (33, 34) because gene data is absent in the NCBI (Takashima et al., unpublished). Homologs listed in Table S5 were used for Fig. 5.

### Statistical analysis

Statistical differences of *mee* gene expression between the parent and a deletion mutant strain were calculated using one-way ANOVA, and those of the parental strain treated with rapamycin were calculated using *t*-test. The *P*-values are significant at *P* < 0.05.

## Supplementary Material

Reviewer comments

## Data Availability

RNA-seq data set of all the samples has been deposited in the DNA Data Bank of Japan (DDBJ) and is accessible with BioProject number PRJDB40648.

## SUPPLEMENTAL MATERIAL

Supplemental tables spectrum.03459-25-s0001.pdf Tables S1 to S6.

OPEN PEER REVIEW reviewer-comments.pdf An accounting of the reviewer comments and feedback.

ASM does not own the copyrights to Supplemental Material that may be linked to, or accessed through, an article. The authors have granted ASM a non-exclusive, world-wide license to publish the Supplemental Material files. Please contact the corresponding author directly for reuse.
