# Supplementary material for: Magnesium enhances the expression of arthroconidia-related genes in Trichosporon asahii
Source: Microbiol Spectr. 2026 Jun 15;14(7):e03459-25. doi: 10.1128/spectrum.03459-25 (PMC13340095; doi:10.1128/spectrum.03459-25)
Supplement: Supplemental tables — Tables S1 to S6. [file spectrum.03459-25-s0001.pdf]

Table S1

KEGG pathway

Category	Term	Genes	Count	List Total	Pop Hits	Pop Tota	P-Value	Benjamini	Fold Enrichment	Bonferroni	FDR	Fisher Exact
KEGG_PATHWAY	Biosynthesis of amino acids	4%	30	221	99	1827	1.6E-06	0.000161	2.51	0.000161	0.00016	5.03E-07
KEGG_PATHWAY	Spliceosome	3%	25	221	81	1827	1.11E-05	0.000562	2.55	0.00112	0.000557	3.42E-06
KEGG_PATHWAY	Biosynthesis of secondary metabolites	7%	54	221	323	1827	0.00637	0.132	1.38	0.476	0.131	0.00425
KEGG_PATHWAY	Pentose phosphate pathway	1%	8	221	20	1827	0.0065	0.132	3.31	0.483	0.131	0.00139
KEGG_PATHWAY	Carbon metabolism	3%	20	221	88	1827	0.00656	0.132	1.88	0.485	0.131	0.00298
KEGG_PATHWAY	2-Oxocarboxylic acid metabolism	1%	10	221	39	1827	0.0373	0.628	2.12	0.979	0.622	0.0144
KEGG_PATHWAY	Motor proteins	1%	8	221	30	1827	0.06	0.767	2.20	0.998	0.76	0.022
KEGG_PATHWAY	Lysine biosynthesis	1%	5	221	13	1827	0.0608	0.767	3.18	0.998	0.76	0.0141
KEGG_PATHWAY	Phenylalanine, tyrosine and tryptophan biosynthes	1%	6	221	19	1827	0.0684	0.767	2.61	0.999	0.76	0.0204
KEGG_PATHWAY	Carbon fixation by Calvin cycle	1%	6	221	20	1827	0.0826	0.81	2.48	1.00e+0	0.802	0.0262
KEGG_PATHWAY	Terpenoid backbone biosynthesis	1%	5	221	15	1827	0.096	0.81	2.76	1.00e+0	0.802	0.0268
KEGG_PATHWAY	Pyrimidine metabolism	1%	7	221	27	1827	0.0963	0.81	2.14	1.00e+0	0.802	0.0365

Table S2

Spliceosome

	Locus_tag	Function
1)	A1Q1_05657	20 kDa nuclear cap binding protein (ncbp)
2)	A1Q1_06890	Pre-mRNA splicing factor RNA helicase PRP28
3)	A1Q1_02769	Pre-mRNA-splicing factor CLF
4)	A1Q1_07633	RNA binding protein
5)	A1Q1_00233	BUD31 family, bud site selection-related protein
6)	A1Q1_07347	cell wall organization and biogenesis-related protein
7)	A1Q1_07196	cyclophilin-like peptidyl prolyl cis-trans isomerase
8)	A1Q1_03133	formin binding protein 3
9)	A1Q1_00713	glycine rich protein
10)	A1Q1_02068	heat shock protein
11)	A1Q1_01227	hypothetical protein
12)	A1Q1_01664	hypothetical protein
13)	A1Q1_03355	hypothetical protein
14)	A1Q1_04638	hypothetical protein
15)	A1Q1_04952	hypothetical protein
16)	A1Q1_05041	hypothetical protein
17)	A1Q1_05956	hypothetical protein
18)	A1Q1_07187	hypothetical protein
19)	A1Q1_01666	mRNA processing-related protein
20)	A1Q1_03525	mago nashi domain-containing protein
21)	A1Q1_04085	nuclear mRNA splicing protein
22)	A1Q1_07352	pre-mRNA splicing factor
23)	A1Q1_07590	pre-mRNA splicing factor
24)	A1Q1_01502	small nuclear ribonucleoprotein E
25)	A1Q1_06149	splicing factor

Motor proteins

	Locus_tag	Function
1)	A1Q1_04634	actin lateral binding protein
2)	A1Q1_00826	hypothetical protein
3)	A1Q1_07121	hypothetical protein
4)	A1Q1_07456	hypothetical protein
5)	A1Q1_00311	kinesin
6)	A1Q1_07208	microtubule motor
7)	A1Q1_04366	myosin, light chain 2, 20 kDa
8)	A1Q1_05428	nonmuscle myosin heavy chain b

Pyrimidine metabolism

	Locus_tag	Function
1)	A1Q1_03909	NADP(+)-dependent 3-hydroxy acid dehydrogenase
2)	A1Q1_03687	carbamoyl-phosphate synthase (glutamine-hydrolyzing)

3)	A1Q1_05150	dCMP deaminase
4)	A1Q1_02187	hypothetical protein
5)	A1Q1_04436	UMP-CMP kinase subfamily
6)	A1Q1_06612	hypothetical protein
7)	A1Q1_01996	uracil phosphoribosyltransferase

Terpenoid backbone biosynthesis

	Locus_tag	Function
1)	A1Q1_04001	farnesyltranstransferase
2)	A1Q1_02236	hypothetical protein
3)	A1Q1_07704	hypothetical protein
4)	A1Q1_06791	hypothetical protein
5)	A1Q1_04488	protein-S-isoprenylcysteine O-methyltransferase

Table S3

Primers used for RT-PCR in the study

Locus tag	Primer F (5' to 3')	Primer R (5' to 3')
A1Q1_04050	TCAAACTTCCCCAACACAGC	GTACACACCCTCCACCACAC
A1Q1_07823	GTCATTTTACCCTCGCCGAATC	TTGGCACTGTCGTAGCTTCTC
A1Q1_07651	CTTTGGTGGTATCTTCGGCTAC	CGGCAATAACGAACGTCAAC
A1Q1_03805	AACTCGATCTCAGCCCATCC	GCCGTCCAGTCTTTCGTTTAG
A1Q1_04659	ACGAAGGGCAAGAAAGTCAG	CAGAGATACTTCAGCGTCGTC
A1Q1_01131	TATGCCATCTTCCTCCCTTGTG	TCAACGCCACCATGTTTGAG
A1Q1_05458	ACTTCACCCTGATCTCAAGCG	ATGTCCGAGTCTGTGAACTGC
A1Q1_01385	GTCAAGGACAAGTACGACATCG	TCCTCTGGTGTCCACATACTG
A1Q1_02761	TGCACTGATTTCGGGGAGAG	GCCCTTCTGTTTCTCGACTTTC
A1Q1_03141	TTTTCGTCGACATGCCCATC	GCCAATCGGAGAGACAACTG
A1Q1_05074	AGCCTGTGAGCTAATTGTCCAG	TCGGCATCATCTTGAGGTTCTC
A1Q1_07116	ATTCAGAAGCTCGTCAAGGC	TCTGCTCCACGTTGATGTTC
A1Q1_05377	AGTGAAGATGAACGCGACAG	TTGGCCATTGTCTTGTGGAC
A1Q1_00560	TCTCGCAACGTCTGATCAAG	TCGCATGTCTCGTCGATTTC
A1Q1_01176	TTCTGCGTTATTCCCGAACC	ACACCAAGAGCAGCCATTG
A1Q1_08064 (actin II)	TCCGCAAAGCAGGTCATTAC	TCGGGTTGAGGGAAAGGTAAC

Table S4

Primers used for gene deletion in the study

Locus tag	Gene	Primer types	Primer 1 (5' to 3')	Primer 2 (5' to 3')
A1Q1_04050	*mee1*	5' untranslated region	CAGAGCAGAACTCGTCAGCCAATA	AGGGTATTCTGGGCCTCCATGTCGCGAAGCGTTGAGCTTAAACTGGCC
		3' untranslated region	GTTCTCGGCAAGTTGTGACCAGCG	ATGTGAATGCTGGTCGCTATACTGGCGCCAGGGAGCGGCGTATACACT
		Check primer F	GTAGCATCACCAGCACTACG	TAAGCCGTGTCGTCAAGAGT
		Check primer R	GCTCCTCAAGACCACATCTG	GCTCTACATGAGCATGCCCT
A1Q1_07651	*mee2*	5' untranslated region	GTGGAGACTGTGACATGGTGACAG	AGGGTATTCTGGGCCTCCATGTCGGTGTCTGTCCTCGGGGTGTCGTCT
		3' untranslated region	CGAGCTCATTTCGCCCTACATCCT	ATGTGAATGCTGGTCGCTATACTGCTGGTGCGGTTCCCACTATCTTTC
		Check primer F	CTTGCTCGTGGGTAAGTGTT	TAAGCCGTGTCGTCAAGAGT
		Check primer R	CAACTTTCTGGATCCGCAGA	GCTCTACATGAGCATGCCCT
A1Q1_03805	*mee3*	5' untranslated region	CTTGTCAGCGCCAGCCATCTCGTC	AGGGTATTCTGGGCCTCCATGTCGCAAGTGGAAGTGTGTGTGCTCGTG
		3' untranslated region	GTTGTTCGACGAGTTTAGGCGTGT	ATGTGAATGCTGGTCGCTATACTGGTCTCCTCTCGCTGACGCGCTGCC
		Check primer F	GAGACGCTTGTGAGCATGCT	TAAGCCGTGTCGTCAAGAGT
		Check primer R	GCATATCAGTAAACTCATGG	GCTCTACATGAGCATGCCCT
A1Q1_01385	*mee4*	5' untranslated region	CAAGTTTGTCACTGTGCACGTCCA	AGGGTATTCTGGGCCTCCATGTCGGCGGTTACTTTCGAGTGCTAGTGG
		3' untranslated region	GGCCGATTACACATCCATTCAGCG	ATGTGAATGCTGGTCGCTATACTGCTAGGAGTGTCATGCATACAACAG
		Check primer F	GAGCTGTTCCGTAACGAGCT	TAAGCCGTGTCGTCAAGAGT
		Check primer R	GACTACGCCTGTGCCAAGAA	GCTCTACATGAGCATGCCCT
A1Q1_02761	*mee5*	5' untranslated region	GTCAGTGACGCAATTTCGGAAATC	AGGGTATTCTGGGCCTCCATGTCGGTTGAGTCGCTTCTGGAGCTGGAC
		3' untranslated region	CTTCACTCGTAACCGCATCAAGAC	ATGTGAATGCTGGTCGCTATACTGCAACATGTTCATATGTATCTATGC
		Check primer F	GTGTATGGTGAGAGTTCGAG	TAAGCCGTGTCGTCAAGAGT
		Check primer R	GAAGTCTGCGGCTCAACTTG	GCTCTACATGAGCATGCCCT
A1Q1_03141	*mee6*	5' untranslated region	GACACGTCCCTCATCATCAACAAC	AGGGTATTCTGGGCCTCCATGTCGCATTGCCCAATGTGTAAACAAGAG
		3' untranslated region	CAGACCAAGCCTGAGCCCGTCTTC	ATGTGAATGCTGGTCGCTATACTGGACTGAGGCTGGATCGAGGACAGA
		Check primer F	CAGTATCCAGCGACCAGCTC	TAAGCCGTGTCGTCAAGAGT
		Check primer R	GGTGCCGAGTCAGAGTCATC	GCTCTACATGAGCATGCCCT
A1Q1_05074	*mee7*	5' untranslated region	GCTGCTTCTCGTGTGGAAAGGTTG	AGGGTATTCTGGGCCTCCATGTCGGGTGGACTGGTTGTTGTTGACCTC
		3' untranslated region	CTGGCTTTGGAAGTTGACTGTAGC	ATGTGAATGCTGGTCGCTATACTGCTCTC CTGTCTCAAT CGCACATTC
		Check primer F	GTGAGTACGAAGTTATCTAG	TAAGCCGTGTCGTCAAGAGT
		Check primer R	CCGAAGAGTCTACTCCACGT	GCTCTACATGAGCATGCCCT
A1Q1_07116	*mee8*	5' untranslated region	CGTCTTGTGCTGGCTCCATATCTC	AGGGTATTCTGGGCCTCCATGTCGGTGTCGTAGTGAGTAAGTGTGAAA
		3' untranslated region	GAAGGTGACTGACTTCGGCTTTGC	ATGTGAATGCTGGTCGCTATACTGGGAGAACGCCAAGTTAGATGTTTA
		Check primer F	CTCTGATAGTGATCGCACTC	TAAGCCGTGTCGTCAAGAGT
		Check primer R	CATTCGCGTAACTGTCTTCA	GCTCTACATGAGCATGCCCT
A1Q1_05377	*mee9*	5' untranslated region	GAGTGCAGACGATACGCGGCTAAG	AGGGTATTCTGGGCCTCCATGTCGGTGAGCGATAGTGTTGAAGGTGGG
		3' untranslated region	CACAATCCAGGGCTCACTGCTGGA	ATGTGAATGCTGGTCGCTATACTGTAAG TGCCGCTACG CCAGGCCGAT
		Check primer F	GATCGGTTGTTGAGAGTCTG	TAAGCCGTGTCGTCAAGAGT
		Check primer R	GTAGATCTTGACAGCATGCG	GCTCTACATGAGCATGCCCT
A1Q1_00560	*mee10*	5' untranslated region	GCTATGAAGGGCTCGTTCTCGATC	AGGGTATTCTGGGCCTCCATGTCGGGCTGTTGATTGCAATGAGGTTGG
		3' untranslated region	GTTGGATTGGATTAGCTGTGAGCC	ATGTGAATGCTGGTCGCTATACTGGGGTCATCTAGTTAGTCAGAAGCG
		Check primer F	CTCCAGGCAAGTTCAAGATG	TAAGCCGTGTCGTCAAGAGT
		Check primer R	CAACAGCTGCCGACTAGATC	GCTCTACATGAGCATGCCCT
A1Q1_01176	*mee11*	5' untranslated region	CTTGCAGAACGCTGCACACCTTGT	AGGGTATTCTGGGCCTCCATGTCGTGTGGGGTGTTGTTGCGGACAAGT
		3' untranslated region	CACTACCATGATCACCACCTCTCT	ATGTGAATGCTGGTCGCTATACTGCATGTCTGCAGGCTCTTAGCTATT
		Check primer F	GTGATGTGAGGTGTGACACA	TAAGCCGTGTCGTCAAGAGT
		Check primer R	CATGGCACTG GTATCACAGT	GCTCTACATGAGCATGCCCT
Nat resistant gene		Check primer K	CGACATGGAGGCCCAGAATACCCT	CAGTATAGCGACCAGCATTCACAT

Table S5

Homologous proteins of Mee4, Mee5, Mee6, and Mee11 were searched using blastp (https://blast.ncbi.nlm.nih.gov/Blast.cgi?PAGE=Proteins).
Amino acid sequences in *T. coremiiforme*, *T. faecale*, and *T. inkin* were examined using OrthoFinder because gene data is absent in the NCBI.

i) *mee4* ortholog used in Fig. 5

Species	Locus_tag	Max score	Total score	Query cover	E-value	Per. Identity	Acc. Length
Trichosporon asahii	A1Q1_01385	233	233	100%	1.00E-76	100	115
Trichosporon coremiiforme	TC1	226	226	100%	1.00E-83	96.52	115
Trichosporon faecale	TF1	229	229	100%	9.00E-85	98.26	115
Trichosporon inkin	TI1	217	217	100%	5.00E-80	92.17	115
Cutaneotrichosporon cavernicola	CcaverHIS019_0504050	209	209	97%	2.00E-67	90.18	118
Cutaneotrichosporon oleaginosum	COLE_01283	202	202	97%	1.00E-64	88.39	118
Vanrija albida	Q8F55_003161	167	167	98%	8.00E-51	73.45	113
Cryptococcus neoformans	CNAG_00840	213	213	98%	1.00E-68	86.73	143
Cryptococcus gattii	CGB_A9540W	215	215	97%	2.00E-69	91.07	143
Cryptococcus amylolentus	L202_07669	183	183	98%	1.00E-56	75.22	144
Kwoniella europaea	V865_007847	215	215	99%	1.00E-69	88.6	121
Kwoniella dendrophila	L201_005239	213	213	99%	7.00E-69	87.72	121
Mycena latifolia	FB451DRAFT_1342863	115	115	91%	3.00E-30	55.24	116
Naganishia albida	NCC49_000157	185	185	98%	7.00E-58	76.11	121
Trametes sanguinea	FKP32DRAFT_1612325	127	127	95%	7.00E-35	55.05	117
Acaromyces ingoldii	FA10DRAFT_304719	194	194	98%	3.00E-61	81.42	123
Pseudomicrostroma glucosiphilum	BCV69DRAFT_284635	189	189	97%	3.00E-59	79.28	122
Rhodotorula toruloides	JCM10021v2_003015	210	210	97%	1.00E-67	89.29	120
Rhodotorula kratochvilovae	JCM10449v2_000483	130	130	98%	5.00E-36	57.89	115
Rhodosporidiobolus lusitaniae	JCM8547_004071	152	152	99%	9.00E-45	66.67	105
Neurospora crassa	B0T13DRAFT_191223	116	116	99%	2.00E-30	51.3	117
Trichoderma virens	TrVGV298_002247	97.4	97.4	92%	5.00E-23	37.74	122
Fusarium graminearum	SNK05_006754	117	117	94%	5.00E-31	50	118
Fusarium decemcellulare	FDECE_3953	132	132	94%	8.00E-37	57.41	118
Sordaria macrospora	SMAC4_01851	113	113	99%	2.00E-29	49.57	118
Botrytis cinerea	BCIN_13g05430	115	115	93%	5.00E-30	53.27	117
Aspergillus fumigatus	KXX03_008356	119	119	94%	8.00E-32	51.85	118
Aspergillus nidulans	ANIA_00331	119	119	94%	2.00E-31	52.78	119
Penicillium chrysogenum	N7489_009982	113	113	94%	3.00E-29	50	118
Dipodascopsis uninucleate	V1511DRAFT_503915	119	119	99%	9.00E-32	48.25	115
Limtongia smithiae	V1518DRAFT_424793	112	112	95%	6.00E-29	48.62	115

ii) *mee5* ortholog used in Fig. 5

Species	Locus_tag	Max score	Total score	Query cover	E-value	Per. Identity	Acc. Length
Trichosporon asahii	A1Q1_02761	365	365	100%	4.00E-127	100	178
Trichosporon coremiiforme	TC2	199	199	100%	1.00E-69	60	246
Trichosporon faecale	TF2	128	128	76%	7.00E-42	62.59	255
Trichosporon inkin	TI2	124	124	48%	2.00E-41	69.77	181
Cutaneotrichosporon cavernicola	CcaverHIS019_0700980	55.1	55.1	51%	6.00E-06	33.33	107
Cutaneotrichosporon oleaginosum	COLE_07767	78.2	78.2	40%	1.00E-14	49.3	115
Kwoniella europaea	V865_001060	55.5	55.5	36%	4.00E-06	40	104
Kwoniella dendrophila	L201_004197	55.1	55.1	36%	9.00E-06	36.92	120

iii) *mee6* ortholog used in Fig. 5

Species	Locus_tag	Max score	Total score	Query cover	E-value	Per. Identity	Acc. Length
Trichosporon asahii	A1Q1_03141	4531	4531	100%	0	100	2254
Trichosporon coremiiforme	TC3	3766	3766	100%	0	90.03	2267
Trichosporon faecale	TF3	3277	3277	99%	0	80.81	2284
Trichosporon inkin	TI3	60.1	60.1	2%	3.00E-12	59.65	2263
Cutaneotrichosporon cavernicola	CcaverHIS019_0102820	720	720	74%	0	36.46	1808
Cutaneotrichosporon oleaginosum	COLE_06630	738	738	72%	0	37.27	1807
Vanrija albida	Q8F55_002861	667	780	57%	0	45.58	1739
Cryptococcus neoformans	CNAG_05343	326	326	13%	7.00E-86	51.35	1520
Cryptococcus gattii	CGB_H0150W	242	242	9%	4.00E-60	53.77	1192

Cryptococcus amylolentus	L202_05744	520	520	19%	2.00E-148	56.26	1741
Kwoniella europaea	V865_007247	504	504	33%	1.00E-141	41.88	2074
Kwoniella dendrophila	L201_000877	508	508	24%	4.00E-142	48.62	2314
Mycena latifolia	FB451DRAFT_1022543	216	216	18%	1.00E-55	33.49	526
Naganishia albida	NCC49_001248	387	387	19%	6.00E-107	45.98	1250
Trametes sanguinea	FKP32DRAFT_1687571	237	237	18%	2.00E-61	36.78	653
Acaromyces ingoldii	FA10DRAFT_284654	273	273	18%	4.00E-69	35.56	1787
Pseudomicrostroma glucosiphilum	BCV69DRAFT_313177	258	258	15%	2.00E-64	37.73	1701
Ustilago hordei	UHO2_03080	292	292	23%	7.00E-75	33.33	1830
Rhodotorula toruloides	JCM10021v2_001460	238	238	18%	4.00E-58	34.65	2091
Rhodotorula kratochvilovae	JCM10449v2_001982	240	240	16%	4.00E-59	38.22	1433
Rhodotorula glutinis	JCM8208_001813	246	246	18%	5.00E-61	36.44	1440
Rhodosporidiobolus lusitaniae	JCM8547_007774	264	264	16%	2.00E-66	41.92	1425

iv) *mee11* ortholog used in Fig. 5

Species	Locus_tag	Max score	Total score	Query cover	E-value	Per. Identity	Acc. Length
Trichosporon asahii	A1Q1_01176	399	399	100%	1.00E-139	100	196
Trichosporon coremiiforme	TC4	193	193	100%	2.00E-67	50	223

>TC1 sequence
MAPTNLVSVNTAPERAKVVIGTVIENVKDKYDIVHAGNSTTIEGVRPLLESVQPLPGILFCASMWTPEQQEEIQRIARETVPGIKTHAIPTGLQVKVGPDGVIK<br>YLMERIDEIMA
>TC2 sequence
MLSSLPLFVQLPLLHTPPRLQRLRHCLSTPSLGDTDDNYHHAAQDHPEATPEKPSPCPSLAWALPRPCMDTLVLLQRKLNMPSNVTWSAAHDTALLTS<br>LVSLILSNRREVYRAPGLSDVANNGGSRINQRLLLMLRKMCTDFGERGLVDELVRAQQAQKPGGARKRKSDKSDGDEEGEERKVKMERVEKVEKNKV<br>KGDKDKGEKDGKGGKGEIAGLSEVEMSAKSKDELRNEILHGNPDVKPSFQY
>TC3 sequence
MAATSSPSRSDGLGMFPNMTPDRSPMYNNFTPPRPSPLRSHPQPQPGPSPSPRHFPSSPQRSSRPPNPSSSYAPGSPTRLPLYQTNPNTPPQKYRVT<br>NPDPPSPLLSDSQSPMQQAQASRAQSPPHRLQLPRAQSPAQQQQQQYRLQQQQQQQQQQQQQQQRRHQQSQSRSAMPPPPQPSFAPAASSPLKP<br>QNGNNRDATSDEMYADASESDEKRSKSSHGSNNSGRGIGLSKSTEFLRNLAGKGKAHAYTELNGEDDDEELLRQPEPTQPKRHSLASLDLRRGSPGS<br>EAPNTAQDIWTGSQEALARGFPQPPHPSSENPYLSDYGFSSQDSFMTAQGSGGATSPIPPVVRGKPWPAHASPQTAGGPQLADPLVPVEGPHSRSDLF<br>AAETGGMLMFDGENFQRGRGLSAASSQAPRPPASDSHSHDHPSSNGSSSRLDFTKPRAEPMPDWNEPAPPLSWVAPADWTSEWSQARNEAQSRRP<br>TRHTRSYSDGMQLLNRQGTLLHPDSSGERQSEELGLMLGKPRNRRLSTGRLLPPPDTSRLKQPEAVRLEASKKRRARVELDVVLERECAVEGGDLRG<br>RLEVVVHGGKRSNTLRVGHGKVRVIGFEEVDGRRHIFYQHQYPLPVFQHWANGPRTTLFADPPDGEGFCAAKEGTHAIPFSIPLPVGEGAKGTYTSHDP<br>KGPNIRYVVVGSVKIFVPKKDKRAIAHFYRPCVILPYLNPAFVLALARAPIMTQRTCGLGWNLTGEKGKVIMQVALGRRIWVAGQRVWCEVAVKNDSNKKI<br>TKCSLAVIQTVNTYLPKKGKQAMSREPTTSHRKKVAEETVEADFMDLGSGHVTGKGWWTGLEPGEGNRWDLSCPIPSGMLSIPRSRYVEVLFTLRVTLN<br>NSVFVDVPIEAINFLSIDPPPMPGDTRRIGGRLPVVSPAGPSLTAGMAGVGAGTAGRSALETSGEGSTLTQMANGNLGTVSSNGGGYDLEQLRGRNATP<br>DADRISNANPSSTTLHIDTIKQNARLSQQDAAASQQASQARTSQPIGEVTEVTEDLAPRPGSPDSQYSTRGGTMASKYSIQSIDEDERNLLASEQARREG<br>RHSIAQTLQIQQLQEEKEKLQRELDALNERRAASQGHPAGAAAAGAVVGSVAGAMAGGKGLETPAVEVTPDESDLPTPTASPQRGMLTVPDDKDEVES<br>VVSGTYAPSHEPKEVSYQMGSDEDDFTDADETPHGSPTHSPAQGPLDTSLPPLQLVPRPLYLSHISEEEEPLDDETLSELMCQDAQHARHIDFGDDDD<br>DLLAEPVSGFLESTPRRSLEARLRSACSPHGDPASPTTPRANSSHCESQEHSASSIGAGLANLNLRAGSPGARDTFGPPSSGHRSTSSVDYTEPIDVED<br>ISMTDMEEEGYDADAVSSEAHGSVYATSPMAAAFQRHQQSQSLPGSWEPSTPWGARRGSRPPALPTLQSARDITWGGVLTGRTVSGRSLKVPFEEEP<br>PLASPSSGTFGTISTGSASNSNGTPSTTRSAHYEHSGLVHDHESRNLAALAEGRDEFAPPRYARTDSVADSANQSFHTAGSSPRSGDSGSGGEQTHMP<br>TLAPSTGSSSGSDGESIPSPRQPEGPHGPEGAFVPNPKDSDAASIPDRFKPKPRVVSGDSMESHMSSVSGVSGMSDMLPSVRARIAALETRDTALRSF<br>GPQSAASSTRVITPQSTGLRPTHTGASLASAGSGLRAASPALRVSRPSTPSEMHLRPISPALTGGSVGSNASGRSSKRKSYTSTLAPRGADSESSQYSM<br>TPNPSQGGHLAVPGSQAEDRDQYSDNESAAEHDDLPRPRSLVEVDDETPRATHQTMFTQMSVPESEADQRASVLTTGTAGTESSSSALRTGGIPPAPI<br>RTSTLSAVSAVDAPTPPKSPVPRGPRPPLRTQTSKPNIIQPQTPTHAASASIPSGMWLSGSPPSGPGALAQDDEDDDSRSSRRYSEIHTVHTFSTEATTP<br>RWSGSTRSSPSKKSDGSPTKPSGAFAKLTGRSPRGTPKRDLSPEPLEGFVRSAQGAYALNTARGSRASLAAPISGDNSGRNSPSNSPLASPRLGALGT<br>VGEHRRNSLQQFIRSGPSSTGHGVSYAESPGYVAEARSPLMVRSATASMEGNIETMSDDEEEVGRKLRSAGQRQLHLTIPPQSMPQAEPVPTPGDGQP<br>QQLHYDPQTESYVPAPAPSEYAAPRSPRSAGSHAPPSPRSAGHSSSGHLQAPLRYGGAYQPGSPKSPTTSTGRLSPHNTDNTDNTGHSSDVSSEWST<br>GYSQVAGRLPRRV
>TC4 sequence
AYSCRLPSQPPSTMFNTLVLAFAAFSAASPALSAYIERPVAFNERDFPVSHLSARGENGTSPSINGSYIVSLIESRVGKQLGGTNNNSTGDLDEGGWPLN<br>CSDQCASFKAFVKDKGLCQVYSIDTNDSSQMTCSGTFKIRASDFCTNLDKAAPCYYCLVPEPSLQSEMKDAFVQDLVVCKKAGVSIPKEVEGLDMSKVS<br>AAYALSISAAVAVTALGTSLLMLA
>TF1 sequence
MAPVNLVSVNTAPERAKKVIGTVIENVKDKYDIVHAGNSTTIEGVKPLLESVQPPPGILFCASMWTPEEQEEIQRIARETVPGIKTHAIPTGLQVKVGPDGVI<br>QYLMERIDEIMA
>TF2 sequence
RGHIRPLQCPHPLLPFFILSLSILLHSYTLPCLPTSRRSSLGDSVRQCTTLSLRHHEDEPQTQASRSQRERRKRHSAPEPPSPAPLPQPRVDTLVPLQRK<br>MPSNVTWTAAHDTALLTSLVSLLLANRREIYRAPGLADVANNGGSRINQRLLLMLRKVCTDFGERGLVDELVRGQAQKTGGRKRKSDESPEGLQGEGK<br>KVKSEKGEKKEVVVKSEKKDMGEKSEKSQQSAQSAQSAQSAQSLAEAMELEVMPKLE
>TF3 sequence
MTSSDPSPMYNNFATPPRPSASASALHQQQQPGPSPSPRHFPSPPRSSRPANPGSTYYAPGSPTRLPLYQTNPNTPPQRYRVTNPDPPSPLLSDSHS

PMQQAQASARQPLPRAQSPHHRQQLPRAQSPAQQQQQQYRLQQQRRQQQSLSVMPPPPQPSFAPAASSPLKPQRDATSDEMYADASESDEKRSKS
SHGSNNSGRGVRMSKSTEFLRNLAGKGKAHAYTELNGEDDDEELLRQPSPVKPKRHSLASLDLNRGSPGLDTARSNWSGSKEALARGFPQPPRGAE
NPYLSDHGFSSQDSFTTAQGSGGATSPIPPVMRARPWPAQASPQTAGGPQIGDPLVPVEGPHSRSDLFAAETGGMLMFDGETFQKGRGPSAASSQFVR
PPASDSHSSDHPSSNSSRLDFTKPRAAPMPDWNEPAPPMSWVAPADWTSEWSQRDAGSRRPTRHARSYSDGIQLLNRQGTLLHPDSSGERLSEELG
LMLGKPRNRRLSTGRLLPPPDTSKLKKPEPVRLEASKKRRARVELDVVLERECAVEGGDLRGRLEVVVHGGKRSNTLRVGHGKVRVIGFEEVDGRRHI
FYQHQYPLPVFQHWPNGPRTTLFADPPDGEGFCAAKEGTHAIPFSIPLPVGEGAKGTYTSHDPKGPNVRYVVVGSVKIFVPKKDKRAIAHFYRPCVILPYL
NPAIVLAPARAPIMTQRTCGLGWSITGEKGKVLMQVALGRRIWVAGQRVWCEVAVKNDSNKKITKCSLAVIQTVNTYLPKKGKQAMSREPTTSHRKKVAE
EVVEADFMDLGSGHVTGKGWWTGLEPGEGNRWDLSCPLPSGMLSIPRSCYVEVLFTLRVTLNNSVFVDMPIEAINFLSIDPPPMPGDTRRIGGRLPVVS
PIGPASLAAGAASAAGLAGVGAGVGGLGASGESSTLTQMANGNLGTVSSNGGGYDLDQLRGRNSTPDADRISNANPSSTTLHIDTIQQNARLSQQGAAA
QTPTQPAIGEVTEVTEDLAPRPGSPDSQYSTRGGTMASKYSVQSVDEDERNLLVSEQARRHGKESIAQKLAIQQLTEEKEKLQRELEAANQRNAERSNL
PAGAAAGVVAGGIAGFVAGGAKGDGAPAVEVTPDESDLPTPTASPSRTPSGMLAVPHDDDAESVVSGTYAPSHEPKEVSYQMGSDEDQEFTDAEDDG
DTPHGSPSLGSTTFSNSQDSLGSLDNSLPPLQLAPRPLYLSHISEEEEPLDDETLSELMSEDAQHARHIDFGDDDDDLLAEPAFAESTPRRSLEDRLRS
ACSPMNDPESPSTPRASSQEDHSSMSAGLANLSLRAGSPGARDTFGPPSSGHRSTSSVDYAEPIDVEDISMTDMEEEGYDADAVSSEAHGSVYATSP
MVAAFQRHQQTRSLPGSWEPSSSSSWEQAPRRGSRPPALPTLQSARDITWGGVLTGRTVSGRSLKVPFEEEPPLASPSSGTFGTISTGSASNSNGTPS
TTRSAHYDHTGHSGIVHDHESRNLAALAEGRDEEELMPPRYGRTDSANTSFHTAGSSPRSPGSGSGSSGERKMPTLAPSTGSSSGSDGESIPSPRQPP
ETQGPVYALPLPHPPKEAKEKDSDAASIPDRFRPRARVASGDSMESHMSSVSGVSGMSDMLPSVRARIAALETRDTALRSFGPQSAASSTRVITPQSTG
LRPTHTGASLASMGSGLRSASPGLRPVSPGGSQLRSRPSTPSEVHLRPISPALTGGSVSSNASSRKRKSYTSTLAPRGSESSQYSMTPNPSQGGHLSV
PRDREDRSAYSESESELEPPRPRSFMEEDETPRASHQTTFTQMSVPESESDQRASVLTTGTSSSGSTLRAGGIPPAPIRTSTLSVASAGGIEAPTPPKSP
VPRGPRPPLRTQTSKPNMAQSGSQPQPGSAQAQSSSASQAQSGSTSHAHSASIPSGMWLSGSPTTPSGPAAFAQDEDDSPDSRRFSEIHTVHTFSTEA
TTPRWSGSTRSSPSKKSDGSPTKPSGAFAKLTGRSPRATPKREVSPEPLDSFARSAQGAYALNTARGSRASLAAPISEDNSGRNSPSNSPLASPRIGAL
GTVGEHRRNSLQQFIRSGPSSTGHGVSYAEGPGYVAEARSPLMVRSATASMEGNIETMSDDEEEVGRELRSAGQRQLHLAIPAQEPRVPSPGDGQPQ
QLHYDPQTESYVPAPAPAEYAARPRSAGSHSAGSHAPPSPRSPAPPSPRSSGSHAPPSPGRAPPSPRGLGSLLTAPLRYAGSYVPGSPKSGRLSPHD
TGNTGHSSDVSSEWSTGYSAVAGRLPRRV

>TI1 sequence

MPAVNLVSVNTAPERAAKVIGTVIENVRGKYDIVHAGNSTTIEGVKPLLESVQPPPGILFCASMWTPEEQEEIQRIARETVPGIKTHAIPTGLQVKVGPDGIV
RYLMERIDEIMA

>TI2 sequence

DPHDRSTHSTHPRPLAGAWCAIRRAVRGCTHRTHRPTSPSSLLPIPSLLVSRSFWICQPRECSTQATMSSPSPVKKPAAPGATWTAAHDCAVLHSLVSL
VLANRREIYKNPALADVANNGGSRINQRILLMLRKMCNDFGEKGLVDELVRAQSAAGRRKRKATDDEDDKREIKPKLEPAQI

>TI3 sequence

MAATSSPSRSDGLGGMFAPDHQRNTMYGYNSFNSPPRSSPLRSHPQPGPSPSPRHFPSSPQRSSRPAHPGASLQATGSPTRLSFYQTTPNTPSPQRY
RVTNPDPPSPVQQSFTLRPNLSGPQPPEHQQQQQQQLLQSPRQRQQQQQRAQSAAQQQQQQQQLQQHYRLPQHQQQQQQQQQALSVMSQPQPSF
APAAPSPLKNHSEAIFNRPQATSDEMYADASESDEMPRSRNSNGSNGASGGRALRMSRSTEFLRNLAGKGKGHAYTELAGEDEDEELLREPAVSRVK
RSSPGSGSPVGNDASAIGFPEQPVRLPTNDNPYSNDHGYSSQDSFTTAHGSGVASGAASPMSPGAPVGRARSRPWPAQASPQTTPAPRAEDPSNPH
GPHSRSALYAAETGGMLMFDGERFQRGRGPSAASSQLAPAPPSDTNSSANHSSNGSSNRPDFTKPRAVPMPNWNEPGPPLSWVAPGDWSSEWREQ
RNDGQARRPTRHARSYSDGVQLLNRQGTLLHPESSGERLSEELGLMLGKPRNRRLSTGRLLPPPDASKLKKPEQVRLEASKKRRARVELDVVLEREC
VVEGGDLRGRLEIVVHGGKRTNTLRVGHGKMRVIGFEEVNGLRHIFYQHQYPLPVFQHWENGPRTTLFGDAPDEEGFCVAKEGTHAIPFSIPLPVGEGAK
GTYTAHDPKGPNVRYVVVGSVKIFVPKKDKRAIAHFYRPCVVLPYLNPAIVLSPARAPIMTQRTCGLGWSIGGEKGKVLMQVALGRRIWVAGQRVWCEV
AVKNDSNKKINKCSLAIIQTVNVFMPKKGKQAMSREPTTSHRRKVAEETVEADFMDLGSGHVTGKGWWTGLEPGEGNRWDLSCPIPGGMLSISRSRYV
EVLFTLRVTLNNSIFVDMPIETINFLSIDPPPMPNDTRRVGGRMPILAQPPDSVFAASGGSLNQMQPAIGESTTLGRMVDGNLGTVSTAGGGYDLDMLRR
AGQTPERDADRISDANPSSTTLHIDAIAQNARMQESEAAALAAQTRERAAVPTITEDLAPRPGSPDSQYSTLRGERSNVQTVASRDGTRDVFSDSGHSV
DEDERNLLESEQARREGHASIAQTLASEKEKQEAEAAAAAAAAAALTMPALASQPESETPTPVASPQPASAPTMTTLAVPGDDKDELESVISERLPEEER
KTYAPAHDPKEVEYDMAASQASDDDEYTDAEDEEPTPHGSPTFPPLQMAPRPLYLSHISEEEEPLDDETLSELMSEDAHHGRYVTCVNEDDDDLLAEQ
PGESGIVESTPRRSLEDRLRSACSPQPGDRPSSPTTPRAVSSQGEHSSGGTAAGVTAALARLAVEGHAGSPGDRDTFGPGPSSSGHRSTSSIDYTEPID
VEDISMTDIEDVQDNDNDVGYDADAVSSQGHGSSYATSPMAAAFQRHVQSRSLPGSWEPAQRPPALPQPESARDITWGGVLTGRTVSGKSLKIPFDHL
DDPPLASPSSGTFGTISHASSSASNSNGTPSSTNKSQLYEGPSQLVHDHESRNLAALAEGREEYGDFELVPPRFTRVDSAAQSFHTANSHSPSSGSGS
SADHHRNVPALAPSTGSSSGSDNESIHSPPTQSQPVYSLPLPPPPPGARSPTTEQTATIPERFKPKPRVVSDDSVESHMSSASVMSGEMLPSVRARIAA
LETRDTALRSFGVASAASSTRVLSPQSTGHGLKPTHTGASLGSGGSHGLRTSSPGPSSLRPVSPSGHLRPASPSAGSRPSSPSQSLLRPISPAHTGGSA
SSNSSSRSKRKSYTAALAPRASPASSASRYSMTPNPSSMGAHLALPSQGQNVPDDDRSMYSHDNESFDDHAPPRSLQEPEDETPRAAHQTMFQHVN
VPDSADARSSVMTTGTYTSESSSSTVRVHTGIPPAPINTQRTSTLSVVTDAPAPPKSPVPRGPRPPLRTQRSNQNMAQHAPSTPIPSGMWFSGQRNDD
DDAESRRSSRPHSEVQTVHTFSTEPTTPRWSGSTRSSPSKKSSEPSPSKPGMSQGTEPSGAFAKLTGHSPRTTPKRSLTPESLEGFARGAQGAYLLNT
AGSRGSRVSVEPEGSAEGSSGGSPLASPRLGAFGTVGEHRRNSLSQFIRAPSSSGHGVSFAESPGSVAETRSPVVVRSVAASMEGQIGTMSVEADSDG
EDEVGRSLRERSPYAPQQQQQPRSQYAQPSALFSQLAPKEAHQQQQQQQQQHRPARLQAPAQYSGGYEPSSPTQGKLSPHSTGTGHSSDVSSDWST
GYSAVAGRLPPRRF

Table S6

Each average value represented in Fig. 6A is shown.

Target gene	cDNA	First PCR	Second PCR	Third PCR	Average	Standard error	*p*-value
actinII	Parent	1.00E+00	1.00E+00	1.00E+00	1.00E+00	0.00E+00	ND
	Δ*mee4*	1.00E+00	1.00E+00	1.00E+00	1.00E+00	0.00E+00	ND
	Δ*mee5*	1.00E+00	1.00E+00	1.00E+00	1.00E+00	0.00E+00	ND
	Δ*mee6*	1.00E+00	1.00E+00	1.00E+00	1.00E+00	0.00E+00	ND
	Δ*mee11*	1.00E+00	1.00E+00	1.00E+00	1.00E+00	0.00E+00	ND
mee4	Parent	3.45E+00	3.40E+00	3.46E+00	3.44E+00	1.81E-02	ND
	Δ*mee4*	ND	ND	ND	ND	ND	ND
	Δ*mee5*	3.23E+00	4.03E+00	3.62E+00	3.63E+00	2.31E-01	$p = 0.9$
	Δ*mee6*	6.45E+00	5.76E+00	5.22E+00	5.81E+00	3.56E-01	$p = 0.007$
	Δ*mee11*	5.94E+00	4.49E+00	6.45E+00	5.63E+00	5.89E-01	$p = 0.01$
mee5	Parent	6.39E+00	9.98E+00	1.10E+01	9.12E+00	1.40E+00	ND
	Δ*mee4*	8.94E+00	1.06E+01	1.04E+01	9.98E+00	5.30E-01	$p = 0.9$
	Δ*mee5*	ND	ND	ND	ND	ND	ND
	Δ*mee6*	8.67E+00	1.04E+01	7.86E+00	8.98E+00	7.63E-01	$p = 0.9$
	Δ*mee11*	2.50E+01	3.98E+01	2.03E+01	2.84E+01	5.87E+00	$p = 0.009$
mee6	Parent	2.08E+00	2.18E+00	2.31E+00	2.19E+00	6.39E-02	ND
	Δ*mee4*	4.51E-01	5.19E-01	5.14E-01	4.95E-01	2.21E-02	$p = 0.001$
	Δ*mee5*	4.54E-01	4.80E-01	4.76E-01	4.70E-01	8.19E-03	$p = 0.001$
	Δ*mee6*	ND	ND	ND	ND	ND	ND
	Δ*mee11*	3.57E+00	3.46E+00	3.45E+00	3.49E+00	3.84E-02	$p = 0.001$
mee11	Parent	8.40E+00	1.04E+01	1.06E+01	9.80E+00	7.15E-01	ND
	Δ*mee4*	3.45E+00	4.04E+00	3.85E+00	3.78E+00	1.76E-01	$p = 0.001$
	Δ*mee5*	9.01E+00	7.18E+00	7.09E+00	7.76E+00	6.26E-01	$p = 0.1$
	Δ*mee6*	8.85E+00	8.55E+00	1.01E+01	9.17E+00	4.61E-01	$p = 0.8$
	Δ*mee11*	ND	ND	ND	ND	ND	ND

The *p*-values were calculated by one-way ANOVA because three samples were compared per a gene.

Each ratio represented in Fig. 6B is shown.

Target gene	cDNA	First PCR	Second PCR	Third PCR	Average	Standard error	*p*-value
mee4	Sab+YNBΔMg	1.00E+00	1.00E+00	1.00E+00	1.00E+00	0.00E+00	ND
	Sab+Mg	3.89E+00	4.05E+00	4.06E+00	4.00E+00	5.50E-02	ND
	Sab+Mg+Ra	3.30E-01	3.52E-01	4.09E-01	3.64E-01	2.35E-02	$p < 0.001$
mee5	Sab+YNBΔMg	1.00E+00	1.00E+00	1.00E+00	1.00E+00	0.00E+00	ND
	Sab+Mg	8.31E+00	7.23E+00	1.49E+01	1.01E+01	2.38E+00	ND
	Sab+Mg+Ra	2.42E+00	2.20E+00	3.85E+00	2.82E+00	5.15E-01	$p = 0.02$
mee6	Sab+YNBΔMg	1.00E+00	1.00E+00	1.00E+00	1.00E+00	0.00E+00	ND
	Sab+Mg	2.68E+00	2.80E+00	3.45E+00	2.97E+00	2.39E-01	ND
	Sab+Mg+Ra	2.31E+00	2.25E+00	2.82E+00	2.46E+00	1.80E-01	$p = 0.08$
mee11	Sab+YNBΔMg	1.00E+00	1.00E+00	1.00E+00	1.00E+00	0.00E+00	ND
	Sab+Mg	3.99E+00	6.91E+00	3.71E+00	4.87E+00	1.03E+00	ND
	Sab+Mg+Ra	1.21E+00	1.33E+00	9.43E-01	1.16E+00	1.15E-01	$p = 0.01$

Sab, Sabouraud medium. Mg, Magnesium. Ra, Rapamycin. YNBΔMg, Yeast nitrogen base without $MgSO_4$.

The *p*-values were calculated by *t*-test because two samples were compared per a gene.
