# Supplementary material for: Magnesium enhances the expression of arthroconidia-related genes in Trichosporon asahii
Source: Microbiol Spectr. 2026 Jun 15;14(7):e03459-25. doi: 10.1128/spectrum.03459-25 (PMC13340095; doi:10.1128/spectrum.03459-25)
Supplement: Reviewer comments [file reviewer-comments.pdf]

AMERICAN SOCIETY FOR MICROBIOLOGY | Microbiology Spectrum

Magnesium enhances the expression of arthroconidia-related genes in *Trichosporon asahii*

Keita Aoki, Minenosuke Matsutani, Moriya Ohkuma, Takashi Sugita, Yuuki Kobayashi, Naoto Tanaka, and Masako Takashima

Corresponding Author(s): Keita Aoki, Tokyo Nogyo Daigaku

Review Timeline:

Submission Date:	October 23, 2025
Editorial Decision:	January 6, 2026
Revision Received:	January 20, 2026
Editorial Decision:	February 18, 2026
Revision Received:	February 20, 2026
Editorial Decision:	February 26, 2026
Revision Received:	April 4, 2026
Editorial Decision:	April 7, 2026
Revision Received:	April 11, 2026
Accepted:	April 27, 2026

Editor: Matthew Anderson

Reviewer(s): The reviewers have opted to remain anonymous.

Transaction Report:

(Note: With the exception of the correction of typographical or spelling errors that could be a source of ambiguity, letters and reports are not edited. The original formatting of letters and referee reports may not be reflected in this compilation.)

DOI: https://doi.org/10.1128/spectrum.03459-25

Re: Spectrum03459-25 (Magnesium enhances the expression of arthroconidia-related genes in *Trichosporon asahii*)

Dear Dr. Keita Aoki:

Thank you for the privilege of reviewing your work. Below you will find my comments, instructions from the Spectrum editorial office, and the reviewer comments.

Both reviewers felt the study was well organized and the data convincing. The points to address are often procedural but remain critical for conveying the results presented here. Please pay special attention to the use of the terminology in the manuscript and inclusion of technical details that are not as complete as they could be.

Please return the manuscript within 60 days; if you cannot complete the modification within this time period, please contact me. If you do not wish to modify the manuscript and prefer to submit it to another journal, notify me immediately so that the manuscript may be formally withdrawn from consideration by Spectrum.

Revision Guidelines
To submit your modified manuscript, log into the submission site at https://spectrum.msubmit.net/cgi-bin/main.plex. Go to Author Tasks and click the appropriate manuscript title to begin. The information you entered when you first submitted the paper will be displayed; update this as necessary. Note the following requirements:

• Upload point-by-point responses to the issues raised by the reviewers in a file named "Response to Reviewers," NOT in your cover letter.
• Upload a compare copy of the manuscript (without figures) as a "Marked-Up Manuscript" file.
• Upload a clean .DOC/.DOCX version of the revised manuscript and remove the previous version.
• Each figure must be uploaded as a separate, editable, high-resolution file (TIFF or EPS preferred), and any multipanel figures must be assembled into one file.
• Any supplemental material intended for posting by ASM should be uploaded with their legends separate from the main manuscript. You can combine all supplemental material into one file (preferred) or split it into a maximum of 10 files with all associated legends included.

For complete guidelines on revision requirements, see our Submission and Review Process webpage. Submission of a paper that does not conform to guidelines may delay acceptance of your manuscript.

Data availability: ASM policy requires that data be available to the public upon online posting of the article, so please verify all links to sequence records, if present, and make sure that each number retrieves the full record of the data. If a new accession number is not linked or a link is broken, provide Spectrum production staff with the correct URL for the record. If the accession numbers for new data are not publicly accessible before the expected online posting of the article, publication may be delayed; please contact production staff (Spectrum@asmusa.org) immediately with the expected release date.

Publication Fees: For information on publication fees and which article types are subject to charges, visit our website. If your manuscript is accepted for publication and any fees apply, you will be contacted separately about payment during the production process; please follow the instructions in that e-mail. Arrangements for payment must be made before your article is published.

ASM Membership: Corresponding authors may join or renew ASM membership to obtain discounts on publication fees. Need to upgrade your membership level? Please contact Customer Service at Service@asmusa.org.

The ASM Journals program strives for constant improvement in our submission and publication process. Please tell us how we can improve your experience by taking this quick Author Survey.

Thank you for submitting your paper to Spectrum.

Sincerely,
Matthew Anderson
Editor
Microbiology Spectrum

Reviewer #1 (Comments for the Author):

The manuscript entitled "Magnesium enhances the expression of arthroconidia-related genes in Trichosporon asahii", by Aoki K and colleagues, presents an important discussion on the role of magnesium in driving hyphal formation, a topic highly relevant to

fungal virulence. The authors have contributed substantially to this field over the past decades, and the results presented here have a notable impact on our understanding of T. asahii, particularly by linking morphogenesis, magnesium signaling, and fungal pathogenesis. However, several important points require attention, as outlined below.

There is a long-standing and well-established convention regarding the term dimorphism, which is traditionally reserved for organisms that (i) exhibit two well-defined morphological forms and (ii) undergo a temperature-dependent morphological transition. In this context, the use of the term "dimorphism" in relation to this yeast is incorrect according to the classical definition of fungal dimorphism. T. asahii does not undergo a temperature-dependent morphological transition, i.e., it lacks the classical thermal switch. For example, both in the abstract and in lines 54-55, the authors state: "Dimorphism in T. asahii consists of three distinct morphological phenotypes: yeast, hyphae, and arthroconidia." Dimorphism, by definition, cannot consist of three phenotypes, as this directly contradicts the classical concept. Therefore, please replace "dimorphism" throughout the manuscript with pleomorphic, polymorphic, or morphological phenotypes, as appropriate.

Additionally, please avoid the expression "remains unexplored" when partial studies already exist, as acknowledged by the authors themselves.

The text requires careful grammatical revision, as there are typographical and agreement errors, for example: "Arthroconidia is" → Arthroconidia are, "remain unknown" → remains unknown, Extra parentheses (e.g., "Shioya-MS Co., Ltd., Hyogo, Japan))").

Finally, please avoid excessive repetition of the phrase "magnesium addition".

The results are strong, well organized, and convincing, showing a clear logical progression. Nevertheless, there are minor terminological issues and some interpretative overstatements that should be adjusted.

The authors report that approximately 10% of genes are affected by magnesium supplementation and propose that increased expression of splicing-related genes may contribute to morphological changes. While this observation is intriguing, the causal relationship between enhanced splicing activity and morphogenesis remains speculative and should be interpreted more cautiously.

Line 99-100: "The difference between Sabouraud broth and Sabouraud + YNBΔMg broth was attributed to YNB components other than $MgSO_4$."
Was this experimentally tested or inferred? If inferred, please consider revising to: "was likely attributable to YNB components other than $MgSO_4$."

Line 104: "annotated in NCBI" - please specify whether this refers to genome annotation.

Lines 107-112: These are interesting results, and it would greatly improve the manuscript if the authors included a sentence providing biological context explaining why these pathways may be related to morphogenesis.

Line 137: "gaps in the cell wall at the edges" - do you mean cell wall discontinuities or regions of reduced electron density? Please clarify.

Lines 164-165: Please adjust the p values for Δmee4 and Δmee5.

Lines 174-175: "not affected much" - please clarify or quantify this statement.

Line 243: "Sabouraud (4% glucose and 1% bacto peptone)" - it is unclear whether this is expressed as w/v. Please clarify.

Lines 242-247: It is not clear whether all media used were broth, agar plates, or both, and at which experimental steps. Please clarify.

RNA-seq assays: The methodological description is incomplete. Essential information is missing, including: sequencing platform (which Illumina system?), read length, single-end or paired-end design, number of reads per sample, and number of biological replicates.

Real-time PCR: The reference gene used for normalization is not mentioned.

Lines 335-339: The identity cutoff (%), minimum coverage, and criteria used to define orthology versus homology are unclear and should be specified.

Why was 4.15 mM $MgSO_4$ chosen? Please clarify and provide an appropriate reference.

Minor points

Line 25 and throughout the text: please correct typographical errors such as "Trichoaporon asahii".

Lines 26-27: "deep-seated mycosis" → deep-seated mycoses (also in line 53).

Lines 32-33: please indicate the total number of genes identified.

Line 116: "confirmed by real-time PCR" → validated by real-time PCR.

Line 16: "NCBI website" → NCBI databases.

"Centrifugated" → centrifuged.

Avoid excessive repetition of "magnesium addition".

Line 313: "5-3, 5-4, and 5-5" - please clarify.

"Number of white farinose colonies were counted" → was counted.

"lied downstream" → lay downstream.

Reviewer #2 (Comments for the Author):

See attached.

The manuscript by Aoki *et al*., explores the transcriptional response to magnesium by *T. asahii*. This work follows up on prior findings from this group that identified magnesium affects hyphal formation. Here, the authors show that there's a large number of genes associated with the presence of magnesium that has connections to morphological states of the cells. Some genes strongly induced by magnesium regulated formation of arthroconidia formation. Four genes led to reduced conidial formation and expression of these genes was tested in the mutant backgrounds of the other genes to put together a putative pathway that included the influence of rapamycin. Altogther, this study is a solid step forward in understanding this cellular response and its association with cell identity.

MAJOR COMMENTS

Figure 3: The relevance of white farinose colonies needs to be explained in the text. This experiment also needs to be performed more than once to show reproducibility consistent with changes relative to WT. There's no discussion of an increased in formation of these colonies. What does an increase suggest?

Lines 136-136: There's no evidence in Figure 4B to prove division by fission v. budding. I would either remove this statement, provide time laps microscopy in support of it, or cite the relevant literature. I do agree that there is a defect in production of these cells in the mutants.

Table 1: Additional details are needed. The categories labeled "RT-PCR" and "Deletion" need added explanations. Numerical values for qRT-PCR measurements should also be included with associated p-values.

mee4 expression is increased in the Δ*mee6*. This is not noted on lines 162-169. The model in Figure 5C would also need to be adjusted since it currently suggests activation by Mee6 towards *mee4*.

All data points must be shown for the data presented. This is an issue throughout the figures. Figure 5 does not include error bars or all data points.

To compare gene expression since multiple tests are being considered, a one-way ANOVA should be used here.

MINOR COMMENTS

Line 94: edit as "…Sabouraud broth**, and** Sabouraud broth supplemented…"

A stylistic suggestion: The Venn diagram is Figure 1C is accurate, but the overlapping regions are not proportional to the number of genes. I would suggest an upsetter plot here, which will provide a better representation of the data.

Figure 2: "Carbon metabolism" is misspelled.

Line 107: The “pathways” described in Figure 2 are more categories since some terms are too broad to be considered any pathway(s), like “Biosynthesis of secondary metabolites”.

Line 460: edit as “…**is** represented…”

The statement on Lines 197-199 should be softened. This is speculative currently as there’s no references to demonstrate increased expression of myosin genes increases cell elongation.

Line 214: edit as “…despite **it**…”

Line 225-227: The lack of arthrospores in the *mee4* and *mee5* mutants may similarly suggest they are most upstream in the process. This would be consistent with a potential role for the spliceosome, which will produce pleiotropic effects.

January 20, 2026

Prof. Matthew Anderson
Editor
Microbiology Spectrum

Dear Prof. Anderson,

These are our point-by-point responses to the comments and suggestions raised by Reviewer 1 and Reviewer 2. Bold-type text indicates the comments from the Reviewers.

To Reviewer 1

The manuscript entitled "Magnesium enhances the expression of arthroconidia-related genes in Trichosporon asahii", by Aoki K and colleagues, presents an important discussion on the role of magnesium in driving hyphal formation, a topic highly relevant to fungal virulence. The authors have contributed substantially to this field over the past decades, and the results presented here have a notable impact on our understanding of T. asahii, particularly by linking morphogenesis, magnesium signaling, and fungal pathogenesis. However, several important points require attention, as outlined below.

There is a long-standing and well-established convention regarding the term dimorphism, which is traditionally reserved for organisms that (i) exhibit two well-defined morphological forms and (ii) undergo a temperature-dependent morphological transition. In this context, the use of the term "dimorphism" in relation to this yeast is incorrect according to the classical definition of fungal dimorphism. T. asahii does not undergo a temperature-dependent morphological transition, i.e., it lacks the classical thermal switch. For example, both in the abstract and in lines 54-55, the authors state: "Dimorphism in T. asahii consists of three distinct morphological phenotypes: yeast, hyphae, and arthroconidia." Dimorphism, by definition, cannot consist of three phenotypes, as this directly contradicts the classical concept. Therefore, please replace "dimorphism" throughout the manuscript with pleomorphic, polymorphic, or morphological phenotypes, as appropriate.

- ***Response:*** We appreciate the Reviewer’s accurate comments about the

definition of dimorphism. To present the morphological phenotypes in *T. asahii* including yeast, hyphae, and arthroconidia, the terms "dimorphism" was replaced with "pleomorphism" at all instances of its use in the revised manuscript.

Additionally, please avoid the expression "remains unexplored" when partial studies already exist, as acknowledged by the authors themselves.

- ***Response:*** Thank you for pointing it out. The sentence was replaced with "the transition between these morphologies is not fully understood" on line 62 in the Introduction section.

The text requires careful grammatical revision, as there are typographical and agreement errors, for example: "Arthroconidia is" → Arthroconidia are, "remain unknown" → remains unknown, Extra parentheses (e.g., "Shioya-MS Co., Ltd., Hyogo, Japan))").

- ***Response:*** The first two among the above-mentioned grammatical errors were revised on lines 58 and 70 in the Introduction section. Regarding extra parentheses in the phrase "Sabouraud-hp (4% glucose and 1% hipolypepton (Shioya-MS Co., Ltd., Hyogo, Japan))," the second set of parentheses were replaced with square brackets, as typically used for nesting on line 277 in the Materials and Methods section.

Finally, please avoid excessive repetition of the phrase "magnesium addition".

- ***Response:*** Thank you for this suggestion. We have either changed or removed the phrase "magnesium addition" from the text at as many instances as possible.

The results are strong, well organized, and convincing, showing a clear logical progression. Nevertheless, there are minor terminological issues and some interpretative overstatements that should be adjusted.

- ***Response:*** Thank you for your valuable advice. In the revision process, we have corrected terminological issues and interpretative overstatements as much as possible. Please check these corrections in the revised manuscript.

The authors report that approximately 10% of genes are affected by magnesium supplementation and propose that increased expression of splicing-related genes

may contribute to morphological changes. While this observation is intriguing, the causal relationship between enhanced splicing activity and morphogenesis remains speculative and should be interpreted more cautiously.

- ***Response:*** We appreciate the Reviewer's interest in the increase in spliceosome factors and discussion. Spliceosome-related genes whose expression increases with magnesium supplementation in *T. asahii* include A1Q1_00233 that belongs to the BUD31 family. Repression of BUD31 leads to decreased filamentation in *Candida albicans*, possibly due to altered intron retention. Thus, it may be common that alteration in splicing activity affects hyphal growth. Furthermore, a relationship between spliceosome and motor proteins has been reported in *Ustilago maydis*. In *U. maydis*, Num1, a homologue of SPF27 that is a component of an integral subunit of the spliceosome, is known to be required for polarized hyphal growth. Num1 interacts with not only spliceosome components Prp19 and Cef1, but also a motor protein Kinesin I and factors of endosome-trafficking processes. Thus, Num1 has dual functions for splicing in the nucleus and Kin I-dependent intracellular trafficking processes in the cytoplasm. Therefore, BUD31 homolog might contribute to kinesin dynamics in *T. asahii*. We have added this discussion on lines 207-218 in the Discussion section.

Line 99-100: "The difference between Sabouraud broth and Sabouraud + YNBΔMg broth was attributed to YNB components other than $MgSO_4$."
Was this experimentally tested or inferred? If inferred, please consider revising to: "was likely attributable to YNB components other than $MgSO_4$."

- ***Response:*** To experimentally test the effect of YNB components other than $MgSO_4$, a mixture of YNB components other than $MgSO_4$ (YNBΔMg) was prepared by self and added to Sabouraud medium. To facilitate better understanding by the readers, this comment has been added on lines 282-284 in the Materials and Methods section.

Line 104: "annotated in NCBI" - please specify whether this refers to genome annotation.

- ***Response:*** Genome annotation was not performed in *T. asahii*. The contigs sequenced were not aligned. To avoid misunderstanding, "annotated" was changed to "predicted" on line 104 in the Results section.

Lines 107-112: These are interesting results, and it would greatly improve the manuscript if the authors included a sentence providing biological context explaining why these pathways may be related to morphogenesis.

- ***Response:*** The putative relationship between spliceosome and motor proteins are written above. Magnesium-induced pyrimidine metabolism might contribute to hyphal formation through synthesis of uridine diphosphate (UDP) and cytidine diphosphate (CDP) because A1Q1_04436, whose expression increases with magnesium supplementation, is a candidate homologue of UMP/CMP kinase (EC 2.7.4.14). Nucleotide diphosphate sugar is a donor to produce carbohydrate chains in glycosylation necessary for cell wall glucan. In *Aspergillus nidulans*, loss of UDP-galactofuranose causes abnormal hyphal morphology. Therefore, magnesium might affect glycosylation, thereby influencing cell wall synthesis and hyphal growth in *T. asahii*. While on terpenoid backbone biosynthesis, synthesis of farnesol might increase with magnesium and relate to hyphal formation. This is because the inhibition of farnesylation of CENP-E, a centromere protein, results in the alteration of the microtubule-centromere interaction and attenuates microtubule extension. We have added the discussion on lines 219-230 in the Discussion section.

Line 137: "gaps in the cell wall at the edges" - do you mean cell wall discontinuities or regions of reduced electron density? Please clarify.

- ***Response:*** It refers to cell wall discontinuities. We added the phrase on line 137 in the Results section.

Lines 164-165: Please adjust the p values for Δmee4 and Δmee5.

- ***Response:*** We adjusted notation of the *p* values for Δ*mee4* and Δ*mee5* between the text and Fig. 6 on lines 165-166 in the Results section.

Lines 174-175: "not affected much" - please clarify or quantify this statement.

- ***Response:*** We appreciate your comment regarding the ambiguous expression. In the revised manuscript, *mee6* expression was unaffected by rapamycin ($p = 0.08$) because ratio values but not original values were used to estimate significance in lines 179-180 in the Results section. In addition, the sentences about other gene expressions were revised. The decrease in

the expression of *mee4*, *mee5*, and *mee11* by rapamycin was quantified as follows. Expression of *mee4*, *mee5*, and *mee11*—which were upregulated by magnesium addition—was decreased by rapamycin to 0.09-fold ($p < 0.001$), 0.3-fold ($p = 0.02$), and 0.2-fold ($p = 0.01$), respectively, on lines 176-178 in the Results section.

Line 243: "Sabouraud (4% glucose and 1% bacto peptone)" - it is unclear whether this is expressed as w/v. Please clarify.

- ***Response:*** The contents of media were calculated using the mass volume percent concentration. Thus, "w/v" followed the concentration values as 4% glucose w/v on lines 276-286 in the Materials and Methods section.

Lines 242-247: It is not clear whether all media used were broth, agar plates, or both, and at which experimental steps. Please clarify.

- ***Response:*** Thank you for pointing out the media information. To purify total RNA for RNA-seq and qPCR experiments, the broth of each medium was used. To observe phenotypes of colonies and cell morphologies, agar plates of each medium were used. The explanations were added on lines 284-286 in the Materials and Methods section.

RNA-seq assays: The methodological description is incomplete. Essential information is missing, including: sequencing platform (which Illumina system?), read length, single-end or paired-end design, number of reads per sample, and number of biological replicates.

- ***Response:*** We appreciate the comments regarding RNA-seq methodology. Sequencing was performed using NextSeq 500 with single-end design. Average length and number of reads for each condition was as follows; Sabouraud (340 bp, 17,356,357 reads), Sabouraud+Mg (329 bp, 16,302,470 reads), Sabouraud+YNB (332 bp, 17,956,720 reads), Sabouraud+YNBΔMg (343 bp, 20,101,619 reads), and YPD (325 bp, 15,754,243 reads). Three replicates were used for conditions without YPD, and two replicates were used for YPD. The information has been described on lines 293-294 and lines 304-308 in the Materials and Methods section.

Real-time PCR: The reference gene used for normalization is not mentioned.

- ***Response:*** The sentence "All values determined using Real-time PCR were

normalized with *actin2* expression" was added on lines 323-324 in the Materials and Methods section.

Lines 335-339: The identity cutoff (%), minimum coverage, and criteria used to define orthology versus homology are unclear and should be specified.

- ***Response:*** As the reviewer mentioned kindly, orthologs and paralogs seem to be mixed in each homolog of *mee4*, *mee5*, and *mee6*. Thus, homologs are more suitable than orthologs for expressing the data in Fig. 5. Therefore, the term "ortholog" was replaced with "homolog" throughout the text.

Why was 4.15 mM $MgSO_4$ chosen? Please clarify and provide an appropriate reference.

- ***Response:*** The concentration of $MgSO_4$ was determined in the previous report as it fully induced hyphal growth in *T. asahii* (ref9: Aoki et al. 2023. Microbiol Spectr 11:e0424222). We added the sentence with a reference on lines 281-282 in the Materials and Methods section.

Minor points

Line 25 and throughout the text: please correct typographical errors such as "Trichoaporon asahii".

- ***Response:*** "*Trichoaporon asahii*" was revised as "*Trichosporon asahii*" on line 25 in the Abstract. We have also corrected several other typographical errors throughout the text.

Lines 26-27: "deep-seated mycosis" → deep-seated mycoses (also in line 53).

- ***Response:*** "deep-seated mycosis" was revised to "deep-seated mycoses" on lines 26 in the Abstract and line 54 in the Introduction section.

Lines 32-33: please indicate the total number of genes identified.

- ***Response:*** On line 32 in the Abstract, we have indicated eleven magnesium-enhanced expression (*mee*) genes identified in total.

Line 116: "confirmed by real-time PCR" → validated by real-time PCR.

- ***Response:*** The phrase "confirmed by real-time PCR" was revised as "validated using real-time PCR" on line 116 in the Results section.

Line 16: "NCBI website" → NCBI databases.

- ***Response:*** "NCBI website" was revised to "NCBI database" on line 143 in the Results section.

"Centrifugated" → centrifuged.

- ***Response:*** "centrifugated" was revised to "centrifuged" on line 346 in the Materials and Methods section.

Avoid excessive repetition of "magnesium addition".

- ***Response:*** We have changed the phrase and removed "magnesium addition" from the text as much as possible.

Line 313: "5-3, 5-4, and 5-5" - please clarify.

- ***Response:*** To examine colony formation, cell mixture was sequentially diluted to 5^{-3}, 5^{-4}, and 5^{-5} and spotted in three places on YPD agar plate as given in Fig. 3A, on line 358 in the Materials and Methods section.

"Number of white farinose colonies were counted" → was counted.

- ***Response:*** "Number of white farinose colonies were counted" was revised to "The number of white farinose colonies was counted" on line 360 in the Materials and Methods section.

"lied downstream" → lay downstream.

- ***Response:*** "lied downstream" was revised to "lay downstream" on line 192 in the Discussion section.

To Reviewer 2

The manuscript by Aoki et al., explores the transcriptional response to magnesium by T. asahii. This work follows up on prior findings from this group that identified magnesium affects hyphal formation. Here, the authors show that there's a large number of genes associated with the presence of magnesium that has connections to morphological states of the cells. Some genes strongly induced by magnesium regulated formation of arthroconidia formation. Four genes led to reduced conidial formation and expression of these genes was tested in the mutant backgrounds of the other genes to put together a putative pathway that included the influence of rapamycin. Altogther, this study is a solid step forward in understanding this cellular response and its association with cell identity.

MAJOR COMMENTS

Figure 3: The relevance of white farinose colonies needs to be explained in the text. This experiment also needs to be performed more than once to show reproducibility consistent with changes relative to WT. There's no discussion of an increased in formation of these colonies. What does an increase suggest?

- ***Response:*** We appreciate the comments to make us realize short of explanation about white farinose colonies. To examine what an increase in white farinose colonies suggests, we observed the colonies under the microscopes (Fig. 4). Our results showed that arthroconidia increased in white farinose colonies in the parental strain. Arthroconidia may mainly form white farinose colonies. The result seemed difficult to understand in the text; hence, we have added it to lines 138-139 in the Results and lines 232-233 in the Discussion sections. In addition, a reproduced result was attached in Fig. S1.

Lines 136-136: There's no evidence in Figure 4B to prove division by fission v. budding. I would either remove this statement, provide time laps microscopy in support of it, or cite the relevant literature. I do agree that there is a defect in production of these cells in the mutants.

- ***Response:*** Thank you for pointing out the overinterpreted result. We have removed the statement "Cylindrical cells divided by fission, not budding in the parent strain."

Table 1: Additional details are needed. The categories labeled "RT-PCR" and "Deletion" need added explanations. Numerical values for qRT-PCR measurements should also be included with associated p-values.

- ***Response:*** We appreciate the helpful comments for Table 1. We have added fold-change values with *p*-values for both cases of RNA-seq and RT-PCR in Table 1. The information about whether gene deletion succeeded or not was omitted from Table 1 because it has been mentioned in the text. Ideally, we should provide information about whether candidate genes are essential for readers. However, it is not possible in the case of *T. asahii* because diploid or degron system has not been explored yet. Thus, we cannot exclude the possibility of unsuccessful experiment when a gene-deleted strain was not obtained. We should solve the issues in the future.

mee4 expression is increased in the Δmee6. This is not noted on lines 162-169. The model in Figure 5C would also need to be adjusted since it currently suggests activation by Mee6 towards mee4.

- ***Response:*** Thank you for pointing out the mistake. We have added the sentence "*Mee4* was also increased in Δ*mee6* (p = 0.007)" and "*mee6* negatively regulate *mee4*" on lines 169-172 in the Results section. As the Reviewer has indicated, inhibition of *mee4* expression by Mee6 is probably important for the cascade because inhibition by Mee11 is not common in yeasts other than *T. asahii* and *T. coremiiforme*. Thus, we have added the sentence "Mee6 inhibited the expression of *mee4*, which may be more common among yeasts with arthroconidia, because homologs of the two genes are broadly conserved in basidiomycetes" on lines 254-256 in the Discussion section. Figure 5C was adjusted as "Mee6 inhibited the expression of *mee4*," accordingly.

All data points must be shown for the data presented. This is an issue throughout the figures. Figure 5 does not include error bars or all data points.

- As the Reviewer pointed out, the bar graph was corrected as all data points present throughout the figures with error bars.

To compare gene expression since multiple tests are being considered, a one-way ANOVA should be used here.

- We appreciate the helpful comments for comparing gene expression. Because four independent values were compared per a gene in Fig. 6A, we followed the Reviewer's suggestion and have retested the significance of the differences using one-way ANOVA instead of *t*-test. In addition, since only one out of three values of *mee11* expression in Δ*mee5* was extremely large in Fig. 6A, the real-time PCR experiment of it was reexamined. While in Fig. 6B, we used the significance of the differences using *t*-test because two independent values were compared for a gene.

MINOR COMMENTS

Line 94: edits as "…Sabouraud broth, and Sabouraud broth supplemented…"

- ***Response:*** Thank you for pointing out the grammatical mistake. We have now revised it.

A stylistic suggestion: The Venn diagram is Figure 1C is accurate, but the overlapping regions are not proportional to the number of genes. I would suggest an upsetter plot here, which will provide a better representation of the data.

- ***Response:*** As the reviewer has kindly proposed, we have considered an UpSet Plot to classify genes because an UpSet Plot presents quantities of values accurately. However, we chose a Venn diagram because it saves space in Figure 1C. The Venn diagram data are enough to find out genes whose expression has increased in media of Sabouraud+Mg, Sabouraud+YNB, and YPD compared with media of Sabouraud and Sabouraud with YNBΔMg.

Figure 2: "Carbon metabolism" is misspelled.

- ***Response:*** Thank you for pointing out the misspelling. We have now revised it.

Line 107: The "pathways" described in Figure 2 are more categories since some terms are too broad to be considered any pathway(s), like "Biosynthesis of secondary metabolites".

- ***Response:*** As the Reviewer has mentioned, some categorized groups are functionally ambiguous to be in line with a biological pathway. However, we cannot change the categorized title because it is named by the KEGG. Thus, we have revised the phrase "pathway" to "biological events" in the

text, which provides a broader meaning.

Line 460: edit as "…is represented…"

- ➢ ***Response:*** Thank you for pointing out the grammatical mistake. We have now revised it.

The statement on Lines 197-199 should be softened. This is speculative currently as there's no references to demonstrate increased expression of myosin genes increases cell elongation.

- ➢ ***Response:*** Thank you for pointing out the overinterpreted statement. We have revised "likely accelerate" to "may cause" on line 203 in the Discussion section. Furthermore, we softened the statement as "magnesium addition may simultaneously affect the expression of genes related to both hyphal and arthroconidial formation in *T. asahii* on line 205-206 in the Discussion section."

Line 214: edit as "…despite it…"

- ➢ ***Response:*** Thank you for the grammatical assistance. The sentence was edited to "although it belongs to Trichosporonales."

Line 225-227: The lack of arthrospores in the mee4 and mee5 mutants may similarly suggest they are most upstream in the process. This would be consistent with a potential role for the spliceosome, which will produce pleiotropic effects.

- ➢ ***Response:*** We appreciate valuable discussion. As the Reviewer has indicated, an increase in spliceosome would affect various events. Thus, we have added the sentence " Increase in spliceosome may affect pleiotropic effects on cell morphology" on line 201-202 in the Discussion section.

Re: Spectrum03459-25R1 (Magnesium enhances the expression of arthroconidia-related genes in *Trichosporon asahii*)

Dear Dr. Keita Aoki:

Thank you for the privilege of reviewing your work. Below you will find my comments, instructions from the Spectrum editorial office, and the reviewer comments.

Please note comments by Reviewer 2. Special attention should be paid to the Mee gene organization and the appropriateness of calling the genetic relationships a pathway v. a network.

Please return the manuscript within 60 days; if you cannot complete the modification within this time period, please contact me. If you do not wish to modify the manuscript and prefer to submit it to another journal, notify me immediately so that the manuscript may be formally withdrawn from consideration by Spectrum.

Revision Guidelines
To submit your modified manuscript, log into the submission site at https://spectrum.msubmit.net/cgi-bin/main.plex. Go to Author Tasks and click the appropriate manuscript title to begin. The information you entered when you first submitted the paper will be displayed; update this as necessary. Note the following requirements:

• Upload point-by-point responses to the issues raised by the reviewers in a file named "Response to Reviewers," NOT in your cover letter.
• Upload a compare copy of the manuscript (without figures) as a "Marked-Up Manuscript" file.
• Upload a clean .DOC/.DOCX version of the revised manuscript and remove the previous version.
• Each figure must be uploaded as a separate, editable, high-resolution file (TIFF or EPS preferred), and any multipanel figures must be assembled into one file.
• Any supplemental material intended for posting by ASM should be uploaded with their legends separate from the main manuscript. You can combine all supplemental material into one file (preferred) or split it into a maximum of 10 files with all associated legends included.

For complete guidelines on revision requirements, see our Submission and Review Process webpage. Submission of a paper that does not conform to guidelines may delay acceptance of your manuscript.

Data availability: ASM policy requires that data be available to the public upon online posting of the article, so please verify all links to sequence records, if present, and make sure that each number retrieves the full record of the data. If a new accession number is not linked or a link is broken, provide Spectrum production staff with the correct URL for the record. If the accession numbers for new data are not publicly accessible before the expected online posting of the article, publication may be delayed; please contact production staff (Spectrum@asmusa.org) immediately with the expected release date.

Publication Fees: For information on publication fees and which article types are subject to charges, visit our website. If your manuscript is accepted for publication and any fees apply, you will be contacted separately about payment during the production process; please follow the instructions in that e-mail. Arrangements for payment must be made before your article is published.

ASM Membership: Corresponding authors may join or renew ASM membership to obtain discounts on publication fees. Need to upgrade your membership level? Please contact Customer Service at Service@asmusa.org.

The ASM Journals program strives for constant improvement in our submission and publication process. Please tell us how we can improve your experience by taking this quick Author Survey.

Thank you for submitting your paper to Spectrum.

Sincerely,
Matthew Anderson
Editor
Microbiology Spectrum

Reviewer #2 (Comments for the Author):

I appreciate the work the authors have performed to improve the manuscript.

Some concerns remain and are listed below:

Line 99: Replace "...The difference between Sabouraud broth and Sabouraud+YNBΔMg broth was..." with "The difference between these two clusters was...". The current phrasing suggests a difference between Sabouraud and Sab+YNB Mg.

Line 103-107: This comparison is a comparison of comparisons, which is not clear from the text. It needs to be made clear that the 722 genes are based on differences in Sab+YNB Mg versus the other media when compared to Sab alone.

What farinose colonies are is still not explained when first mentioned. This should be included on line 121.

Line 129-130: Rewrite as "We visualized colonies of the parental strain and the deletion mutants on YPD plates."

Line 138: Revise "...that was cell wall discontinuities..." with "...that showed discontinuities in the cell wall..."

The model of mee regulation in Figure 6C would be improved by not having two Mee11 boxes. This suggests there are two different Mee11 functional units, which there is not evidence for. Please collapse Mee11 into a single representative shape. This change will likely require rewording the relationship of the genes as no clear gene is completely downstream of any other because of feedback among Mee genes. I would suggest calling it an interconnected regulatory network.

February 20, 2026

Prof. Matthew Anderson
Editor
Microbiology Spectrum

Dear Prof. Anderson,

These are our point-by-point responses to the comments and suggestions raised by Reviewer 2. Bold-type text indicates the comments from the Reviewers.

To Reviewer 2

I appreciate the work the authors have performed to improve the manuscript.

Some concerns remain and are listed below:

Line 99: Replace "...The difference between Sabouraud broth and Sabouraud+YNBΔMg broth was..." with "The difference between these two clusters was...". The current phrasing suggests a difference between Sabouraud and Sab+YNBΔMg.

- ***Response:*** Thank you for pointing out the confusing part. The sentence does not focus on the differences between two clusters: the first cluster of YPD broth, Sabouraud+Mg broth, and Sabouraud+YNB broth, and the second cluster of Sabouraud broth and Sabouraud+YNBΔMg broth. The sentence simply represents the difference between Sabouraud broth and Sabouraud+YNBΔMg broth in the analyses of PCA and heat map in Fig. 1.

Line 103-107: This comparison is a comparison of comparisons, which is not clear from the text. It needs to be made clear that the 722 genes are based on differences in Sab+YNBΔMg versus the other media when compared to Sab alone.

- ***Response:*** Thank you for pointing out the ambiguous text. To explain gene expression difference against Sabouraud alone, we added the sentence "Genes whose expression was altered more than 2-fold were 1898 in cells cultivated in YPD, 1680 in Sabouraud+Mg, 1947 in Sabouraud+YNB, and 1423 in Sabouraud+YNBΔMg when compared to Sabouraud alone" in the

line 103-106. Furthermore, we added the sentence “ Thus, 722 genes were identified based on their differential expression between cells cultivated in Sabouraud+YNBΔMg and those in other three media” in the line 106-108 to mention the result clearly.

What farinose colonies are is still not explained when first mentioned. This should be included on line 121.

- ***Response:*** Thank you for pointing out the insufficient explanation. The phenotype of farinose colonies is introduced in the reference 7, the book of “The yeasts, a taxonomic study 5th”. Thus, we added the phrase “that exhibit the powdery surface commonly seen in Trichosporon yeasts” in the line 124-125 to explain farinose colonies.

Line 129-130: Rewrite as "We visualized colonies of the parental strain and the deletion mutants on YPD plates."

- ***Response:*** Thank you for the correction. The sentence was corrected to “We visualized colonies of the parental strain and the deletion mutants on YPD plates” in the line 133.

Line 138: Revise "...that was cell wall discontinuities..." with "...that showed discontinuities in the cell wall..."

- ***Response:*** Thank you for the revision. The phrase was corrected to “...that showed discontinuities in the cell wall...” in the line 139.

The model of mee regulation in Figure 6C would be improved by not having two Mee11 boxes. This suggests there are two different Mee11 functional units, which there is not evidence for. Please collapse Mee11 into a single representative shape. This change will likely require rewording the relationship of the genes as no clear gene is completely downstream of any other because of feedback among Mee genes. I would suggest calling it an interconnected regulatory network.

- ***Response:*** We appreciate the important suggestion. As the reviewer pointed out, our model may mislead readers into thinking that Mee11 has different functional units. Therefore, we propose a new gene expression network in which Mee11 positions in the center of the network in Fig. 6C. In the legend, additional explanations about a new gene expression network were described as “Mee4 and Mee5 were surrounded as a group by a dotted line.

Expression relationships between Mee6 and the group were represented by thick-curved arrow lines" in the line 543-545.

As the reviewer mentioned, the expression regulation of *mee* genes is in the feedback loop. Thus, the phrases of "gene expression cascade" were corrected to the phrases of "gene expression network" in the lines 38, 186, 197, 271, 533, and 543. Even if gene expression regulation is in a network rather than a cascade, Mee4 and Mee5 are thought to act the nearest to arthroconidia formation because white farinose colonies were never observed in Δ*mee4* and Δ*mee5*. Therefore, the phrase of "the most downstream in the pathway of arthroconidia formation" was corrected to "the nearest to arthroconidia formation" in the line 263.

Re: Spectrum03459-25R2 (Magnesium enhances the expression of arthroconidia-related genes in *Trichosporon asahii*)

Dear Dr. Keita Aoki:

Thank you for the privilege of reviewing your work. Below you will find my comments, instructions from the Spectrum editorial office, and the reviewer comments.

All comments from reviewers have been adequately addressed. Prior to acceptance, the data generated from RNA-Seq should be deposited in a publicly available database. Once this is completed, the manuscript should be able to move forward. Please see the Data Availability section below.

Please return the manuscript within 60 days; if you cannot complete the modification within this time period, please contact me. If you do not wish to modify the manuscript and prefer to submit it to another journal, notify me immediately so that the manuscript may be formally withdrawn from consideration by Spectrum.

Revision Guidelines
To submit your modified manuscript, log into the submission site at https://spectrum.msubmit.net/cgi-bin/main.plex. Go to Author Tasks and click the appropriate manuscript title to begin. The information you entered when you first submitted the paper will be displayed; update this as necessary. Note the following requirements:

• Upload point-by-point responses to the issues raised by the reviewers in a file named "Response to Reviewers," NOT in your cover letter.
• Upload a compare copy of the manuscript (without figures) as a "Marked-Up Manuscript" file.
• Upload a clean .DOC/.DOCX version of the revised manuscript and remove the previous version.
• Each figure must be uploaded as a separate, editable, high-resolution file (TIFF or EPS preferred), and any multipanel figures must be assembled into one file.
• Any supplemental material intended for posting by ASM should be uploaded with their legends separate from the main manuscript. You can combine all supplemental material into one file (preferred) or split it into a maximum of 10 files with all associated legends included.

For complete guidelines on revision requirements, see our Submission and Review Process webpage. Submission of a paper that does not conform to guidelines may delay acceptance of your manuscript.

Data availability: ASM policy requires that data be available to the public upon online posting of the article, so please verify all links to sequence records, if present, and make sure that each number retrieves the full record of the data. If a new accession number is not linked or a link is broken, provide Spectrum production staff with the correct URL for the record. If the accession numbers for new data are not publicly accessible before the expected online posting of the article, publication may be delayed; please contact production staff (Spectrum@asmusa.org) immediately with the expected release date.

Publication Fees: For information on publication fees and which article types are subject to charges, visit our website. If your manuscript is accepted for publication and any fees apply, you will be contacted separately about payment during the production process; please follow the instructions in that e-mail. Arrangements for payment must be made before your article is published.

ASM Membership: Corresponding authors may join or renew ASM membership to obtain discounts on publication fees. Need to upgrade your membership level? Please contact Customer Service at Service@asmusa.org.

The ASM Journals program strives for constant improvement in our submission and publication process. Please tell us how we can improve your experience by taking this quick Author Survey.

Thank you for submitting your paper to Spectrum.

Sincerely,
Matthew Anderson
Editor
Microbiology Spectrum

Re: Spectrum03459-25R3 (Magnesium enhances the expression of arthroconidia-related genes in *Trichosporon asahii*)

Dear Dr. Keita Aoki:

No Response to Reviewer was included in this submission. Please return the manuscript within 60 days; if you cannot complete the modification within this time period, please contact me. If you do not wish to modify the manuscript and prefer to submit it to another journal, notify me immediately so that the manuscript may be formally withdrawn from consideration by Spectrum.

Revision Guidelines
To submit your modified manuscript, log into the submission site at https://spectrum.msubmit.net/cgi-bin/main.plex. Go to Author Tasks and click the appropriate manuscript title to begin. The information you entered when you first submitted the paper will be displayed; update this as necessary. Note the following requirements:

• Upload point-by-point responses to the issues raised by the reviewers in a file named "Response to Reviewers," NOT in your cover letter.
• Upload a compare copy of the manuscript (without figures) as a "Marked-Up Manuscript" file.
• Upload a clean .DOC/.DOCX version of the revised manuscript and remove the previous version.
• Each figure must be uploaded as a separate, editable, high-resolution file (TIFF or EPS preferred), and any multipanel figures must be assembled into one file.
• Any supplemental material intended for posting by ASM should be uploaded with their legends separate from the main manuscript. You can combine all supplemental material into one file (preferred) or split it into a maximum of 10 files with all associated legends included.

For complete guidelines on revision requirements, see our Submission and Review Process webpage. Submission of a paper that does not conform to guidelines may delay acceptance of your manuscript.

Data availability: ASM policy requires that data be available to the public upon online posting of the article, so please verify all links to sequence records, if present, and make sure that each number retrieves the full record of the data. If a new accession number is not linked or a link is broken, provide Spectrum production staff with the correct URL for the record. If the accession numbers for new data are not publicly accessible before the expected online posting of the article, publication may be delayed; please contact production staff (Spectrum@asmusa.org) immediately with the expected release date.

Publication Fees: For information on publication fees and which article types are subject to charges, visit our website. If your manuscript is accepted for publication and any fees apply, you will be contacted separately about payment during the production process; please follow the instructions in that e-mail. Arrangements for payment must be made before your article is published.

ASM Membership: Corresponding authors may join or renew ASM membership to obtain discounts on publication fees. Need to upgrade your membership level? Please contact Customer Service at Service@asmusa.org.

The ASM Journals program strives for constant improvement in our submission and publication process. Please tell us how we can improve your experience by taking this quick Author Survey.

Thank you for submitting your paper to Spectrum.

Sincerely,
Matthew Anderson
Editor
Microbiology Spectrum

April 11, 2026

Prof. Matthew Anderson
Editor
Microbiology Spectrum

Dear Prof. Anderson,

We have registered the RNA-seq data with the DNA Data Bank of Japan (DDBJ) and added a "Data availability" section to the manuscript. Since the decision letter for Spectrum03459-25R2 indicated that all reviewer comments have been adequately addressed, no other revisions were made to the manuscript or figures apart from the addition.

Re: Spectrum03459-25R4 (Magnesium enhances the expression of arthroconidia-related genes in *Trichosporon asahii*)

Dear Dr. Keita Aoki:

Your manuscript has been accepted, and I am forwarding it to the ASM production staff for publication. Your paper will first be checked to make sure all elements meet the technical requirements. ASM staff will contact you if anything needs to be revised before copyediting and production can begin. Otherwise, you will be notified when your proofs are ready to be viewed.

Data Availability: ASM policy requires that data be available to the public upon online posting of the article, so please verify all links to sequence records, if present, and make sure that each number retrieves the full record of the data. If a new accession number is not linked or a link is broken, provide production staff with the correct URL for the record. If the accession numbers for new data are not publicly accessible before the expected online posting of the article, publication may be delayed; please contact ASM production staff immediately with the expected release date.

Publication Fees: For information on publication fees and which article types have charges, please visit our website. We have partnered with Copyright Clearance Center (CCC) to collect author charges. If fees apply to your paper, you will receive a message from no-reply@copyright.com with further instructions. For questions related to paying charges through RightsLink, please contact CCC at ASM_Support@copyright.com or toll free at +1-877-622-5543. CCC makes every attempt to respond to all emails within 24 hours.

ASM Membership: Corresponding authors may join or renew ASM membership to obtain discounts on publication fees. Need to upgrade your membership level? Please contact Customer Service at Service@asmusa.org.

Embargo Policy: A press release may be issued as soon as the manuscript is posted on the Spectrum Latest Articles webpage. The corresponding author will receive an email with the subject line "ASM Journals Author Services Notification" when the article is available online.

The ASM Journals program strives for constant improvement in our submission and publication process. Please tell us how we can improve your experience by taking this quick Author Survey.

Thank you for submitting your paper to Spectrum.

Sincerely,
Matthew Anderson
Editor
Microbiology Spectrum
